# Nucleic acid aptamers in orthopedic diseases: promising therapeutic agents for bone disorders

**DOI:** 10.1038/s41413-025-00447-8

**Published:** 2025-07-24

**Authors:** Zhenhong He, Qingping Peng, Wenying Bin, Luyao Zhao, Yihuang Chen, Yuanqun Zhang, Weihu Yang, Xingchen Yan, Huan Liu

**Affiliations:** 1 Department of Orthopedics, The Affiliated Traditional Chinese Medicine Hospital, Southwest Medical University, Luzhou, Sichuan China; 2 Collage of Integrated Traditional Chinese and Western Medicine, Southwest Medical University, Luzhou, Sichuan China; 3 Putian University, Putian, Fujian China; 4 Key Laboratory of Biorheological Science and Technology, Ministry of Education College of Bioengineering, Chongqing University, Chongqing, China; 5 Institute of New Materials, Guangdong Academy of Sciences, State Key Laboratory of Special Materials Surface Engineering, Guangzhou, Guangdong China

**Keywords:** Bone, Bone cancer

## Abstract

Precision medicine has become a cornerstone in modern therapeutic strategies, with nucleic acid aptamers emerging as pivotal tools due to their unique properties. These oligonucleotide fragments, selected through the Systematic Evolution of Ligands by Exponential Enrichment process, exhibit high affinity and specificity toward their targets, such as DNA, RNA, proteins, and other biomolecules. Nucleic acid aptamers offer significant advantages over traditional therapeutic agents, including superior biological stability, minimal immunogenicity, and the capacity for universal chemical modifications that enhance their in vivo performance and targeting precision. In the realm of osseous tissue repair and regeneration, a complex physiological process essential for maintaining skeletal integrity, aptamers have shown remarkable potential in influencing molecular pathways crucial for bone regeneration, promoting osteogenic differentiation and supporting osteoblast survival. By engineering aptamers to regulate inflammatory responses and facilitate the proliferation and differentiation of fibroblasts, these oligonucleotides can be integrated into advanced drug delivery systems, significantly improving bone repair efficacy while minimizing adverse effects. Aptamer-mediated strategies, including the use of siRNA and miRNA mimics or inhibitors, have shown efficacy in enhancing bone mass and microstructure. These approaches hold transformative potential for treating a range of orthopedic conditions like osteoporosis, osteosarcoma, and osteoarthritis. This review synthesizes the molecular mechanisms and biological roles of aptamers in orthopedic diseases, emphasizing their potential to drive innovative and effective therapeutic interventions.

## Introduction

In the pursuit of novel and efficacious therapeutic strategies for disease treatment, scientific researchers are continuously striving for innovation. In recent years, precision medicine has emerged as a pivotal approach in the management of numerous diseases. Notably, nucleic acid aptamer, a closely associated technology, has attracted widespread attention. These aptamers are oligonucleotide fragments derived from the systematic evolution of ligands by exponential enrichment (SELEX) technology, characterized by their high affinity and specificity towards target molecules.^1,2^ They can be either DNA or RNA sequences, with some studies also exploring nucleic acid analogs and peptides. Aptamers possess notable advantages, including considerable biological stability, potential for universal chemical modification, low immunogenicity, and rapid tissue penetration.^3^ In recent years, significant advancements have been achieved in targeted gene therapy research. Current research focuses on delivering therapeutic genes to target cells via specific antibodies or ligands, along with the use of disease-specific gene promoters to tightly regulate gene expression within the disease microenvironment.^3,4^ Although viral vectors demonstrate high efficacy in gene delivery, concerns regarding their safety, immunogenicity, and manufacturing complexities restrict their advancement in clinical applications. In contrast, nonviral gene delivery systems exhibit considerable potential owing to their reduced immunogenicity and simpler manufacturing processes.^5^ Nonviral systems possess a longer shelf-life, lower production costs, and reduced batch-to-batch variability. Moreover, their physicochemical properties and target specificity can be tailored by modifying the nucleic acid composition. They are readily amenable to chemical synthesis and modification to enhance enzyme resistance and in vivo pharmacokinetics.^6^ The incorporation of aptamers into nonviral vectors or their combination with other nonviral vectors can improve the targeting, cellular uptake efficiency, and endosomal escape capability of nucleic acid therapeutics. For example, aptamers can be modified or conjugated with other materials to facilitate improved loading and protection of nucleic acid drugs, thereby enabling more precise delivery to specific cells or tissue sites and achieving enhanced therapeutic efficacy.^6–8^ This review aims to summarize and elucidate the molecular mechanisms and biological roles of nucleic acid aptamers in orthopedic diseases, proposing innovative and effective strategies for future medical interventions.

## The research history and advantages of nucleic acid aptamers

The development of SELEX technology in 1990 marked the emergence of nucleic acid aptamers.^2,9^ Subsequent discoveries included RNA aptamers with catalytic activity in vitro and microRNAs (miRNAs).^10–13^ From the mid-1990s to the early 21st century, research primarily focused on developing aptamers for small molecule sensing applications.^14–16^ A significant advancement occurred in 2001 with the development of aptamer-based protein labeling and detection techniques.^17,18^ The introduction of Cell-SELEX in 2003 was followed by the successful development of the first aptamer-based drug.^19–21^ Further innovations emerged in 2006 with non-covalently coupled aptamer-DOX conjugates and the establishment of aptamer-based optical and electrochemical sensing platforms during the mid-2000s.^15,16,22^ The concept of liquid biopsy gained prominence around 2010, leading to preliminary research on circulating tumor cell release and separation methods using nucleic acid aptamers.^23–25^ Investigations into miRNA detection methods utilizing aptamers commenced in 2015.^26^ During the SARS-CoV-2 pandemic, researchers successfully isolated DNA aptamers targeting the viral spike protein.^27–31^ Recent advancements have focused on developing aptamer-drug conjugates for cancer therapy and diagnostic methods for periprosthetic joint infection (PJI).^32–35^

Nucleic acid aptamers demonstrate enhanced stability through their secondary structures, including stem-loops, hairpins, and G-quadruplexes, with base modifications and stable phosphate backbones contributing significantly to their structural integrity.^36–38^ Protein binding further protects aptamers from external influences.^39^ Environmental factors, particularly ion concentration and pH levels, significantly influence aptamer stability.^9^ Technological advancements have introduced nucleotide analogs to enhance binding affinity and nanotechnology applications for co-delivery with therapeutic molecules.^7,40–43^ Chemical modifications prevent enzymatic degradation and alter binding properties. Optimization of connection modes, length, and density is essential for developing functional aptamer conjugates.^38,42–48^ Multivalent aptamers improve detection sensitivity, while pre-structured DNA libraries expand screening diversity.^7,49–51^ The formation of aptamer-drug complexes through therapeutic agent conjugation improves drug targeting and efficacy.^52–56^ Advanced technologies, including microfluidic technology and high-throughput sequencing, have optimized separation efficiency and the SELEX process.^57–59^

**Fig. 1:**
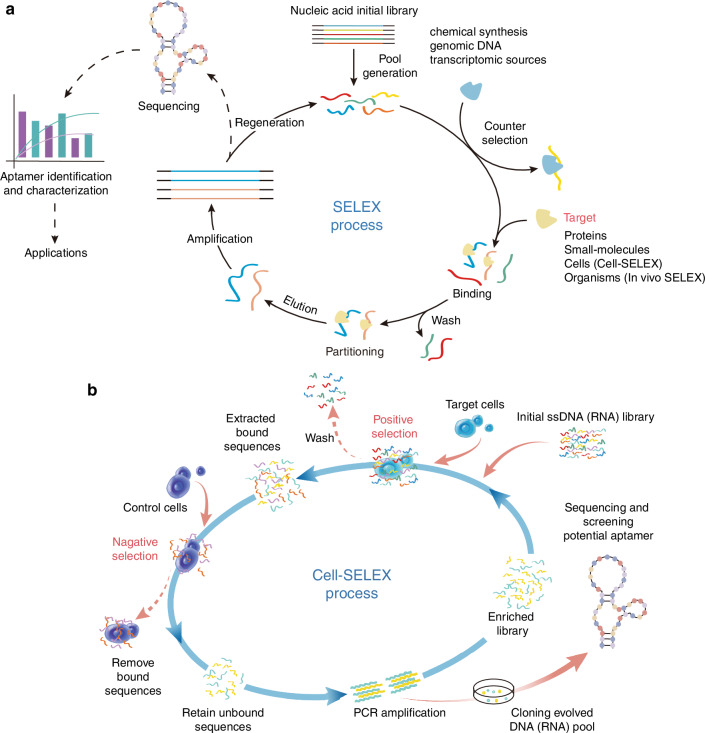
The SELEX process diagram. **a** Schematic representation of SELEX process. **b** Schematic representation of cell-SELEX process

The three-dimensional (3D) conformation of an aptamer, derived from its secondary structure, is pivotal in determining its binding affinity and specificity to homologous targets.^60^ This structural diversity endows aptamers with the remarkable ability to recognize a wide array of target molecules, including proteins, RNA, DNA, and small molecules, with exceptional specificity and affinity.^60–62^ The functionality of aptamer-target complexes is underpinned by a network of intramolecular interactions, such as hydrogen bonds, hydrophobic interactions, van der Waals forces, and complementary base pairing.^62^ These interactions synergistically facilitate the precise recognition and binding of aptamers to their targets.^63,64^ SELEX, a widely utilized method for aptamer screening, has found extensive applications, particularly in the biomedical domain, where it is instrumental in selecting aptamers against proteins, tumor markers, and other biomolecules (Fig. 1a). The aptamers are invaluable in diagnostics, therapeutics, and research due to their high affinity and specificity. Furthermore, SELEX can be refined through various iterations, such as Cell-SELEX (Fig. 1b) and in vivo SELEX, which are tailored for the selection of aptamers in a cell-specific context and within living organisms, respectively.^65,66^ The counter-SELEX strategy introduces a negative selection step to exclude aptamers that bind to non-target molecules, thereby enhancing specificity by distinguishing between highly similar structures.^66,67^ Beyond SELEX, other methodologies can also be employed to augment the specificity and affinity of aptamers. Competitive binding assays, for instance, allow for the assessment of the binding affinity between an aptamer and its target, enabling the optimization of experimental conditions to improve aptamer performance.^68,69^ This approach is particularly useful in selecting aptamers that can effectively compete for target binding sites, thereby enhancing their efficacy in practical applications. In recent years, advancements in separation techniques, such as fluorescence-activated cell sorting (FACS), capillary electrophoresis, solid-phase, and chip-based microfluidic methods, have emerged to refine the affinity and specificity of candidate aptamers.^70,71^ The field of aptamer screening has witnessed the advent of innovative techniques aimed at overcoming the limitations of traditional methods. Particle display technology transforms solution-phase aptamers into aptamer particles, enabling quantitative screening based on affinity through FACS. It effectively separates high-affinity aptamers in fewer selection rounds, minimizing selection bias and human error.^72,73^ Multiparameter particle display further refines this process by employing dual-color FACS to simultaneously evaluate affinity and specificity, facilitating the identification of high-performance aptamers even in complex environments like serum, thus mitigating the risk of discarding potentially valuable aptamers.^73,74^ Click chemistry particle display integrates click chemistry with particle display, simplifying the generation and high-throughput screening of “non-natural” aptamers with extensive base modifications. This innovation addresses the chemical diversity limitations of natural nucleic acid aptamers, opening new avenues for aptamer selection against challenging targets.^73,75^ Collectively, these advancements have significantly propelled the development and application of aptamer technology.

## The applications of nucleic acid aptamers in bone-related diseases

Bone repair and healing constitute a complex and highly coordinated physiological process aimed at restoring or maintaining osseous function.^76^ The biological importance of this process encompasses several aspects. Primarily, bones provide structural support and protection for internal organs, making bone repair essential for maintaining anatomical integrity. Investigation of the cellular and molecular mechanisms involved in this process enhances understanding of general tissue repair and regeneration processes.^76^ With global population aging, the escalating risk of osteoporosis-related fractures underscores the significance of optimizing bone healing to improve geriatric quality of life. Current treatments frequently struggle to completely restore pre-fracture biomechanical properties, particularly in complex open fractures or elderly patients.^77^ Factors such as chronic inflammation, diabetes, vitamin deficiency, aging, and polytrauma can impede healing progression.^78^ Although various technologies and pharmacological agents have been developed to facilitate bone repair and healing, clinical validation remains insufficient for many applications.^79^ Moreover, bone grafting and substitute materials face challenges such as immunogenicity, infection risks, and donor source limitations.^80^

Aptamers exert crucial regulatory effects on bone repair through modulation of key molecular pathways, including parathyroid hormone (PTH), bone morphogenetic proteins (BMPs), and the Wnt/β-catenin signaling pathway.^81–83^ PTH promotes osteogenic differentiation by reducing osteoblast apoptosis and increasing the number of osteoblasts.^84^ Under specific conditions, PTH activates bone lining cell transformation into active osteoblasts.^85–89^ Besides, PTH expands osteoprogenitor cell reservoirs in bone marrow and direct early osteoblast lineage cells away from the adipocyte lineage, thereby promoting their differentiation into osteoblasts.^90,91^ On the other hand, PTH stimulates osteoclastogenesis through non-cell-autonomous mechanisms, enhancing bone resorption to liberate growth factors from the bone matrix that subsequently promote osteoblast migration, differentiation, and functional activation.^92,93^ BMPs synergize with angiogenic factors (eg, VEGF) and metallic cofactors to induce mesenchymal stem cells (MSCs) osteoblastic differentiation while coordinating osteogenesis and angiogenesis during repair.^94–102^

The Wnt signaling pathway can determine the differentiation of mesenchymal precursor cells into osteoblasts or chondrocytes, with β-catenin serving as a vital molecular switch.^103–105^ Concurrently, the expression of key components within the Wnt signaling pathway is regulated during osteoblast differentiation, influencing cellular proliferation, apoptosis, and functional maturation.^81^ Furthermore, the Wnt signaling pathway promotes the differentiation of osteoblasts by inhibiting adipogenic transcription factors CCAAT/enhancer binding protein alpha and peroxisome proliferator-activated receptor-γ (PPARγ).^106,107^ Osteoprotegerin (OPG), a soluble decoy receptor, competitively inhibits receptor activator of nuclear factor-κB ligand (RANKL)–RANK interaction by binding RANKL trimers, thereby blocking osteoclast differentiation and inducing apoptosis.^108^ RANKL, a tumor necrosis factor (TNF) superfamily member upregulated in postmenopausal women, can be primarily modulated by estrogen supplementation.^109^ It stimulates osteoclastogenesis while suppressing osteoclast apoptosis. OPG competes with RANK for binding to RANKL, thus preventing the generation and maturation of osteoclasts induced by RANKL.^110^ Therefore, the OPG/RANKL ratio is intimately associated with osteoclastogenesis, ultimately influencing bone mineral density and mechanical strength. In β-catenin-deficient osteoblasts, RANKL expression is upregulated and OPG expression is downregulated, whereas adenomatous polyposis coli (Apc)-deficient osteoblasts demonstrate inverse regulation.^111,112^ This regulatory network enables Wnt signaling to indirectly govern osteoclast differentiation through osteoblast-mediated mechanisms, thus coordinating bone resorption during repair processes.^113^

### Fracture

**Fig. 2:**
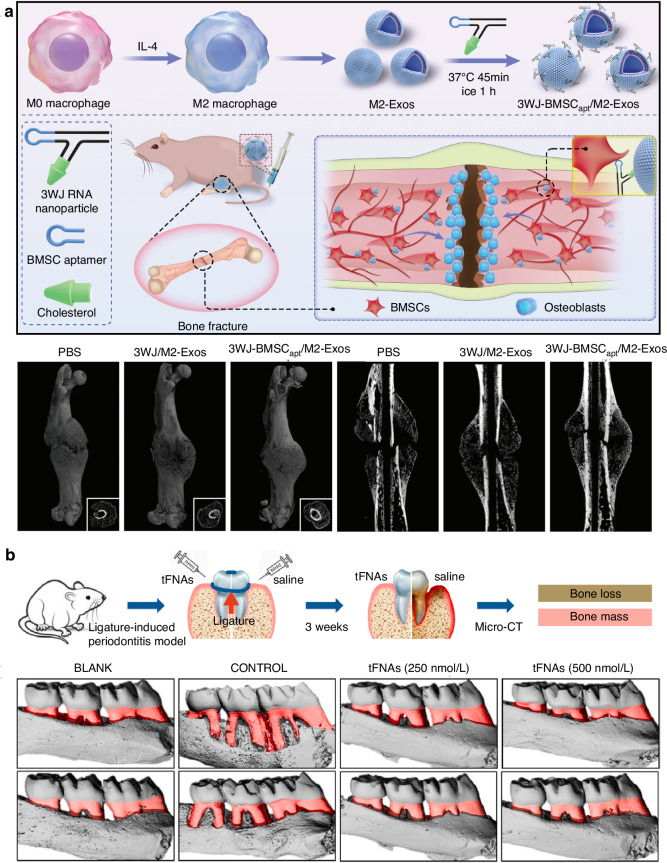
Evaluation of bone repair based on nucleic acid aptamer intervention. **a** Overview of 3WJ-BMSC_apt_/M2-Exos in fracture model. Representative 3D reconstruction images, 2D cross-sectional and sagittal images from micro-CT scanning of the murine femur fracture model. PBS: phosphate buffered saline. Reproduced form ref. ^132^ with permission from Oxford University Press, copyright 2023. **b** tFNAs significantly protected the alveolar bone from periodontitis in vivo. Schematic representation of the rat periodontitis experiment. The micro-CT 3D reconstruction images of the left maxillary alveolar bone. The red area indicates the exposure of the root. Reproduced form ref. ^190^ with permission from KeAi Communications Co, copyright 2020

Fractures refer to pathological conditions characterized by the disruption of bone continuity or integrity due to external forces. These injuries can occur in any bone. The etiology of fractures is multifactorial, including direct or indirect external forces, chronic repetitive stress, and osteoporosis secondary to diseases or medications. Clinically, the healing process of fractures is typically divided into three stages: hematoma formation, callus formation, and callus remodeling. The injury results in the bone breaking into two or more fragments, accompanied by damage to the surrounding periosteum, blood vessels, and other tissues. Extravasation from bone marrow and vasculature leads to the formation of a localized hematoma containing bone-derived and immune cells.^114^ Immediately following a fracture, the inflammatory response is activated, serving as a critical initiator of the bone healing process. The inflammatory period is a crucial stage characterized by hypoxia, impaired perfusion, and the migration of various growth factors.^115^ The fracture hematoma is essential for effective bone regeneration due to its high osteogenic potential.^116,117^ This characteristic was initially observed in non-specialized bone progenitor cells, now identified as MSCs.^118,119^ MSCs are multipotent stem cells that can be isolated from many sources in both animals and humans, such as bone marrow, adipose tissue, amniotic fluid, and periosteum. Bone marrow contains the highest abundance of these cells, which are collectively termed bone marrow mesenchymal stromal cells (BMSCs).^120,121^ These cells possess the potential for osteogenic, chondrogenic, and adipogenic differentiation.^122^ Aptamer-loaded delivery systems can stimulate the osteogenic differentiation of these stem cells, thereby promoting the repair and regeneration of bone tissue.^123^ The fracture hematoma also contains platelets, macrophages, and other cell types.^124^ Cytokines triggering coagulation within the hematoma simultaneously activate local phagocytic cells, facilitating debris clearance at the fracture site.^125^ Successful fracture healing demands coordinated interactions between macrophages and BMSCs. During the process of bone repair, the polarization of macrophages plays a key role in regulating the differentiation of BMSCs. M1 macrophages predominantly mediate pro-inflammatory responses, while M2 macrophages promote tissue repair by enhancing BMSC osteogenesis.^126^ Meanwhile, M2 macrophages are increasingly recognized as positive regulatory factors for fracture healing.^127^ Macrophages facilitate fracture healing primarily through paracrine mechanisms mediated by exosomes.^128,129^ Exosomes derived from M2 macrophages (M2-Exos) serve as key paracrine effectors, driving BMSC osteogenic differentiation.^128,130,131^ Nevertheless, current therapeutic applications of exosomes are limited by nonspecific delivery and rapid clearance by the reticuloendothelial system.^132–134^ Shou et al.^132^ utilized interleukin (IL)-4 to induce the differentiation of the murine macrophage cell line RAW264.7 into M2 macrophages, isolated exosomes (M2-Exos) from osteoblasts, and characterized these exosomes using transmission electron microscopy, nanoparticle tracking analysis, and Western blot. After identifying a BMSC-specific aptamer sequence validated by flow cytometry and confocal microscopy, they engineered 3WJ RNA nanoparticles with three functional components: a_3wj_-Cholesterol, b_3wj_-BMSC aptamer, and c_3wj_-Alexa 647 (Table 1). Chain a was conjugated with cholesterol for anchoring to the exosome membrane, chain b was coupled with the BMSC aptamer serving as a targeting ligand, and chain c was labeled with Alexa 647 for imaging purposes. Chains were mixed at a 1:1:1 molar ratio and self-assembled via gradual cooling from 95 °C to 4 °C at 0.1 °C/s. The resulting 3WJ-BMSC_apt_/M2-Exos system combines BMSC-targeting aptamers with M2-Exos through 3WJ RNA nanoparticles. This functionalized exosome platform demonstrated precise BMSC targeting in vitro and significant fracture site accumulation in vivo, accelerating bone regeneration in a murine femoral fracture model (Fig. 2a). This cell-free strategy offers a novel therapeutic approach, with potential applications in clinically targeted drug delivery. The acute inflammatory response peaks within 24 h and resolves by day 7.^135^ Concurrently, the hematoma transitions to granulation tissue by about day 7.^114^ As inflammation subsides, the repair phase initiates with the formation of granulation tissue.^124^ With the emergence of MSCs and their differentiation into fibroblasts, the hematoma tissue gradually organizes and forms fibrous tissue,^136,137^ known as fibrous callus, which initially bridges the fracture ends.

**Table 1:** Summary of the application of relevant aptamers in orthopedic diseases

Aptamer	Material/Drug	Molecular target	Function	Diseases/Therapeutic effect	Research progress	Ref.
3WJ-BMSC_apt_/M2-Exos a3wj-Cholesterol b3wj-BMSCaptamer c3wj-Alexa 647	Drug	BMSC	Modification of M2-Exos enhances their targeting to BMSCs	In vitro, targeting BMSCs more effectively enhances their proliferation, migration, and osteogenic differentiationIn vivo, it stimulates callus formation, increases bone volume/total volume (BV/TV), bone mineral density (BMD), and trabecular number (Tb. N), reduces trabecular separation (Tb. Sp), promotes new bone formation, and elevates the expression of osteogenic markers ALP and OCN	Under development	^132^
Apt Apt01 Apt02	Material	VEGF receptor	They exhibit high affinity for VEGFR-1 and VEGFR-2	Simulating the function of VEGF-A, it has the potential to activate VEGR receptors and promote angiogenesis	Under development	^159^
Aptamer-agomiR-195	Drug	Endothelial cell	It specifically targets endothelial cells and increases miR-195 levels in cells	Increasing the number of CD31^hi^Emcn^hi^ vessels in aged murines stimulates bone formation and improves bone strength	Under development	^297^
aptamer-antagomiR-188	Drug	BMSC	It regulates the expression of miR-188 in BMSCs	Reduce miR-188 levels in BMSCs to increase trabecular volume, quantity, and cortical bone thickness; decrease trabecular separation and intimal circumference; enhance bone strength; elevate the bone formation rate; reduce the number and area of adipocytes in the bone marrow; and increase the number and surface area of osteoblasts on the trabecular and intimal bone surfaces, thereby preventing bone loss and fat accumulation in the bone marrow of elderly murines	Under development	^298^
OS-7.9	Material	MG-63	It shows high affinity and specific binding to MG-63 cells, as well as recognition of lung and colon colorectal adenocarcinoma cell lines	It enables early diagnosis and targeted treatment of osteosarcoma and metastatic disease	Under development	^317^
SL1067	Material	IL-1α	It demonstrates high affinity and specific recognition of IL-1α	Inhibit the IL-1α signaling pathway to treat related inflammatory reactions and diseases	Under development	^272^
VR11	Material	TNFα	It exhibits high affinity binding and specific recognition of TNFα, inhibiting its receptor binding and blocking TNFα-induced cytotoxicity and NO production	Treat inflammatory diseases and avoid immune reactions	Under development	^273^
8A-35	Drug	IL-8	It shows high affinity binding to IL-8, inhibiting IL-8 receptor binding, regulating the IL-8-induced intracellular signaling pathway, and inhibiting neutrophil migration	Potential anti-cancer effects in treating inflammatory diseases	Under development	^274^
Apc001PE aptscl56	Drug	The loop3 region of sclerostin	It inhibits the antagonistic effect of sclerostin on Wnt signaling pathway and osteogenic potential	It promotes bone formation without increasing cardiovascular risk	Under development	^507^

The primary hematoma undergoes reorganization into granulation tissue, thereby initiating endochondral ossification. This process generates a cartilaginous callus in the endosteum, intramedullary region, and periosteum. Subsequently, the cartilaginous callus is replaced by a hard callus.^138^ Endochondral ossification occurs both between fracture ends and externally in the periosteal region. Proliferating osteoblasts produce ossified tissue, forming new bones. These zones remain mechanically unstable until cartilaginous tissue forms a soft callus that stabilizes the fracture structure.^139^ Intramembranous ossification beneath the periosteum generates new bone adjacent to the fracture ends, contributing to hard callus formation. The bridging of this central hard callus ultimately provides a semi-rigid structure capable of weight-bearing.^140^ The osteoblast-specific aptamer CH6, identified via Cell-SELEX screening, enables the preparation of functionalized lipid nanoparticles encapsulating osteoblast-targeted Plekho1 siRNA (CH6-LNPs-siRNA). Targeted delivery of this siRNA to bone formation surfaces enhances osteogenesis.^67,141–143^ CH6 exhibits high-affinity binding to osteoblasts with minimal interaction with rat osteoclasts, human osteoclasts, or hepatocytes. CH6-LNPs-siRNA possesses excellent in vitro osteoblast selectivity, promoting siRNA uptake while significantly reducing liver/kidney accumulation compared to conventional vectors.^67^ This system achieves osteoblast-specific siRNA delivery, minimizing the uptake of siRNA by liver cells, Kupffer cells, and peripheral blood mononuclear cells.^67,144–148^ As markers of osteoblast activity, alkaline phosphatase (Alp) and osteocalcin (OCN) are closely associated with bone formation.^146,147^ CH6-LNPs-siRNA shows enhanced gene silencing efficiency in Alp^+^/OCN^+^ cells, with Plekho1 mRNA reduction exhibiting dose-dependent and sustained effects that facilitate bone formation.^67^ During callus maturation, hypertrophic chondrocytes undergo extracellular matrix calcification, synergized by macrophage colony-stimulating factor (M-CSF), RANKL, OPG, and TNF-α. These factors mediate mineralized cartilage resorption, osteocyte/osteoclast recruitment, and woven bone formation.^149,150^ TNF-α additionally promotes MSC recruitment and initiates chondrocyte apoptosis.^150^ Aptamer-based delivery systems can regulate osteoclast activity by targeting specific molecules, achieving balanced bone metabolism. This system can simultaneously promote BMSC-mediated bone formation and inhibit osteoclast-mediated resorption. This approach effectively counteracts post-traumatic bone loss such as osteoporosis while improving trabecular microstructure and mechanical properties.^151^

Although hard callus provides biomechanical stability, complete restoration requires remodeling into lamellar bone with a central medullary cavity.^150^ This remodeling phase involves marrow space reconstruction, hematopoietic tissue regeneration, vascular bed normalization, and blood flow restoration to pre-injury levels.^152,153^ The hard callus is progressively remodeled; periosteal and endosteal calluses are absorbed, and the medullary cavity is restored.^150^ As discussed, aptamers targeting osteoblast differentiation factors could accelerate callus formation, while those suppressing osteoclast activity may protect bone integrity. Successful remodeling depends critically on adequate vascular supply and progressive mechanical loading.^154–156^ These principles are extensively validated in fracture studies. VEGF, a hypoxia-inducible angiogenic regulator, plays pivotal roles in vascular development, angiogenesis and fracture healing.^157,158^ Among these, VEGF-A is an essential molecule that activates and binds to VEGF receptor-1 (VEGFR-1) and VEGFR-2. Due to the limitations of recombinant VEGF-A, including low stability, significant batch-to-batch variations, and high production costs, Apt01 and Apt02 (Table 1) have been successfully isolated via SELEX. These aptamers exhibit a high affinity for VEGFR-1 and VEGFR-2, with dissociation rate constants comparable to those of VEGF-A. Notably, their binding affinity hierarchy (VEGFR-1>VEGFR-2) mirrors that of VEGF-A, suggesting DNA aptamers may functionally substitute for VEGF-A.^159^ In summary, aptamer-mediated precision regulation of bone repair processes represents a novel therapeutic paradigm. By orchestrating tissue regeneration pathways, this approach could advance targeted treatments for orthopedic pathologies.

### Alveolar bone

Alveolar bone is the protrusion located on the lower border of the maxilla and the upper border of the mandible, surrounding the root of the tooth. Through the periodontal ligament, it is closely connected to the tooth root and plays a critical role in tooth development, eruption, and mastication processes.^160^ Under pathological conditions including oral inflammatory diseases, trauma, tumors, hereditary diseases, and systemic diseases, the balance between alveolar bone resorption and formation is disrupted, resulting in alveolar bone defects. These defects can cause tooth loosening and loss, trigger masticatory dysfunction, and endanger the physical and mental health of patients.^161^ In dental implant therapy, alveolar bone resorption following tooth extraction or defect leads to insufficient bone volume and altered alveolar ridge dimensions, thereby affecting dental implant placement and complicating treatment.^162,163^ Furthermore, alveolar bone defects can also impact facial appearance, masticatory function, nutrient intake, and cause counterclockwise rotation of the mandible.^164–166^

In the process of alveolar bone repair and reconstruction, signaling pathways including Notch, Wnt, Toll-like receptor, and NF-κB regulate vital activities such as proliferation, differentiation, apoptosis, and autophagy of osteoclasts, osteoblasts, osteocytes, periodontal ligament cells, macrophages, and adaptive immune cells. These pathways also modulate the expression of inflammatory mediators and influence the balance of the RANKL/RANK/OPG system, thereby participating in the repair and reconstruction of alveolar bone.^167–176^ The Notch signaling pathway functions through a complex network of pro-inflammatory cytokines and bone resorption regulators. Notably, Notch1 signaling promotes alveolar bone repair, whereas Notch2 signaling inhibits it.^177,178^ Notch2 upregulation coincides with increased IL-1β and IL-6 levels, creating an osteoclast-favorable environment.^168^ In periapical periodontitis lesions where RANKL expression exceeds OPG, Notch2, Jagged1, Hey1, and TNF-α are overexpressed and strongly correlated, collectively driving extensive alveolar bone resorption.^169^

In a murine periapical periodontitis model, systemic administration of the Wnt inhibitor IWR-1 via the caudal vein significantly exacerbates alveolar bone lesions. Conversely, lithium chloride (a Wnt activator) applied for root canal sealing enhances collagen type I α1 and Runx2 expression, increases CD45R-positive cells, and accelerates alveolar bone healing via bone formation and immune responses.^170^ Furthermore, estrogen activates the Wnt signaling pathway through estrogen receptors, stimulating osteoblast differentiation, inhibiting RANKL-induced osteoclast activity, promoting osteocyte autophagy, and preventing apoptosis, directly contributing to alveolar bone reconstruction. Additionally, estrogen indirectly regulates the metabolic homeostasis of alveolar bone by suppressing oxidative stress and inflammatory responses.^171,172^ TNF-α stimulates gingival MSCs to secrete exosomes rich in miRNA-1260b, which inhibits non-canonical Wnt5a and JNK signaling, downregulates RANKL, upregulates OPG, reduces osteoclastogenesis, polarizes M2 macrophages, and enhances alveolar bone repair in murine periodontitis.^172^ IL-6 activates canonical Wnt signaling via Wnt2b/Wnt10b and non-canonical signaling via Wnt5a, collectively inducing osteogenic differentiation of human periodontal ligament stem cells to promote alveolar bone regeneration.^179^

The NF-κB pathway directly influences inflammatory alveolar bone repair by regulating cytokines and mediators expression. In a ligature and Escherichia coli lipopolysaccharide induced murine periodontitis model, dimethyloxaloylglycine enhances hypoxia-inducible factor-1α expression, suppresses NF-κB phosphorylation, downregulates pro-inflammatory cytokines, upregulates anti-inflammatory cytokines, reduces the M1/M2 macrophages ratio, inhibits osteoclast differentiation, attenuates alveolar bone resorption, and increases alveolar bone volume and density.^174^ Intraperitoneal injection of pirfenidone inhibits the NF-κB signaling pathway in bone marrow-derived macrophages, suppressing the expression of pro-inflammatory cytokines including IL-1β, IL-6, and TNF-α induced by lipopolysaccharide, as well as inhibiting RANKL-induced osteoclastogenesis, which significantly mitigates alveolar bone loss.^175^ NF-κB also indirectly affects bone repair by regulating inflammasome assembly, pro-inflammatory factor maturation and pyroptosis. IL-37 inhibits NF-κB to suppress nucleotide-binding oligomerization domain-like receptor protein 3 inflammasome, reducing osteoclast numbers, calcitonin receptor/RANKL/IL-10 expression, while increasing OPG/IL-1β/IL-6/TNF-α levels, ultimately alleviating alveolar bone resorption.^180,181^

Periodontal ligament stem cells (PDLSCs), a subset of dental pulp stem cells, exhibit unique multi-lineage differentiation into cementum, osteoid, and periodontal ligament-like tissues. This multi-lineage differentiation potential has not been observed in other MSCs, making it a unique characteristic of PDLSCs.^182^ PDLSCs are ideal seed cells for true periodontal regeneration, but inflammatory microenvironments threaten this process by exacerbating tissue destruction and impairing the osteogenic differentiation and migration of PDLSCs.^183,184^ Tetrahedral framework nucleic acids (tFNAs), with unique biological properties, enhance proliferation and osteogenic/odontogenic differentiation of dental pulp stem cells (DPSC)/PDLSC and exert anti-inflammatory/antioxidant effects by inhibiting MAPK phosphorylation in macrophages.^185–189^ Thereupon, Yunfeng Lin et al.^190^ discovered in their research that tFNAs can suppress the release of pro-inflammatory factors in cells, along with the production of cellular reactive oxygen species (ROS), facilitating the migration and osteogenic differentiation of PDLSCs in vitro. It is indicated that tFNAs may exert a protective effect on the osteogenic differentiation of PDLSCs in inflammatory conditions. Furthermore, in the murine periodontitis model, tFNAs reduce inflammatory cell infiltration, downregulate IL-6/IL-1β, inhibit osteoclastogenesis, and protect periodontal tissues (Fig. 2b). Moreover, tFNA activates Wnt/β-catenin signaling to drive proliferation and osteogenic differentiation of PDLSCs. Since its development, tFNA functionalization strategies have been extensively explored. Professor Yunfeng Lin’s team has applied tFNA to orthopedic, metabolic diseases, sarcopenia, bladder obstruction, and cancer,^191–196^ establishing new frameworks for nucleic acid aptamer research. This material offers novel tools for fundamental studies on nucleic acid functions and structure-activity relationships, while holding diagnostic and therapeutic potential for previously intractable diseases.

### Articular cartilage and osteochondral tissue

Articular cartilage is a type of hyaline cartilage primarily composed of proteoglycans and type II collagen within the matrix. It is categorized into deep, middle, and superficial zones.^197,198^ The meniscus is one of the most significant articular cartilages in the human body, playing an extremely crucial position in protecting the health and integrity of the knee joint during long-term weight-bearing activities. Cartilages like the meniscus lack the capacity to generate adequate healing responses independently.^199^ Previously, research on the reconstruction and regeneration of cartilages and osteochondral tissues posed considerable challenges. Yet currently, with the emergence and maturity of new technologies, many of these difficulties are gradually being conquered.

The meniscus can be divided into three different zones: the highly vascularized (red) peripheral region, the avascular (white) inner region, and the red-white transitional region, which possesses characteristics of both.^200^ Meniscus tears are generally recognized to heal poorly due to inadequate vascular supply and reduced cell proliferation.^200–202^ Vascular supply is critical for the healing of the meniscus. The superior, lateral, and medial genicular arteries, branching from the popliteal artery, provide the primary blood supply to the meniscus, forming peripheral capillary plexuses within the synovial and capsular tissues of the knee joint.^202–206^ In adults, vascularization is limited to the peripheral 10%–25% of the meniscus, and the extent of this vascularized area determines the healing potential of tears.^202^ Because of the supply of oxygen, essential factors, and nutrients from adjacent blood vessels, tears in the vascularized area can facilitate tissue healing, while injuries in the avascular region remain irreparable.^207^ Insufficient blood supply leads to deficiencies in nutrients, oxygen, and other critical elements, restricting the repair capacity of the meniscus’s avascular region.^208^ In terms of biomechanics, clinical studies have shown that cartilage damage always extends deeply into the subchondral bone, resulting in osteochondral defects in the knee joint. These defects alter joint biomechanics and impair the long-term performance of cartilage tissue,^199^ underscoring the vital importance of simultaneous articular cartilage and subchondral bone restoration for successful knee joint repair.

Currently, treatment strategies mainly consist of arthroscopic suture, partial or total meniscectomy, and meniscal allograft transplantation (MAT). Nevertheless, arthroscopic meniscectomy inevitably results in progressive cartilage degeneration and osteoarthritis.^209^ While MAT has been clinically implemented, it faces challenges such as donor scarcity, implant shrinkage, and disease transmission risks.^210^ Aptamers are widely used in various biomedical applications, including disease diagnosis, drug delivery, and biosensors, because of their high affinity, specificity, and stability. In meniscus research, aptamers may be utilized to recognize and target specific biomarkers of meniscus injury or degeneration, thereby facilitating early diagnosis and treatment of the condition. Moreover, nucleic acid technologies like PCR and metagenomic next-generation sequencing (mNGS) have demonstrated significant potential in pathogen identification for joint prosthesis infections, with mNGS showing 95% sensitivity and 94.7% specificity in diagnosing PJI.^211–213^ These findings highlight nucleic acid technologies’ diagnostic precision, further exemplified by aptamers’ ability to target and diagnose specific biomarkers.

**Fig. 3:**
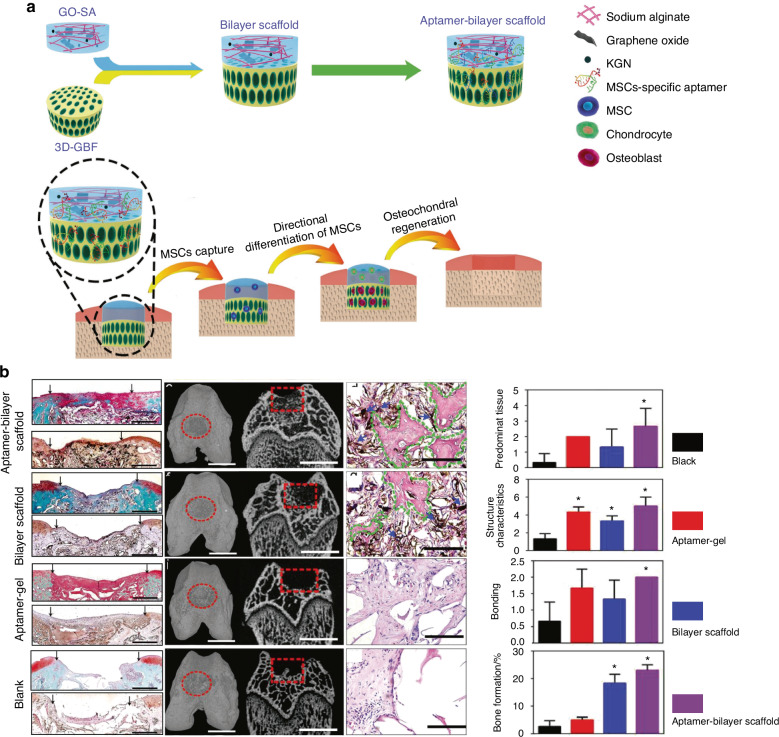
Preparation process of a nucleic acid aptamer-bilayer scaffold and its therapeutic evaluation for osteochondral defects. **a** Overview of the aptamer-bilayer scaffold in osteochondral defect repair. **b** The scaffold was implanted into the osteochondral defect in rats. After an eight-week healing period, histomorphological analyses were performed, including safranin-O staining for glycosaminoglycans (GAG) and immunohistochemical staining for collagen type II to evaluate cartilage regeneration. Concurrently, microcomputed tomography (μCT) reconstruction and hematoxylin and eosin (H&E) staining of articular joint samples were conducted to assess subchondral bone regeneration. The aptamer-bilayer scaffold demonstrated significantly higher scores across all evaluated parameters compared to other groups. Reproduced form ref. ^217^ with permission from Wiley, copyright 2017

**Fig. 4:**
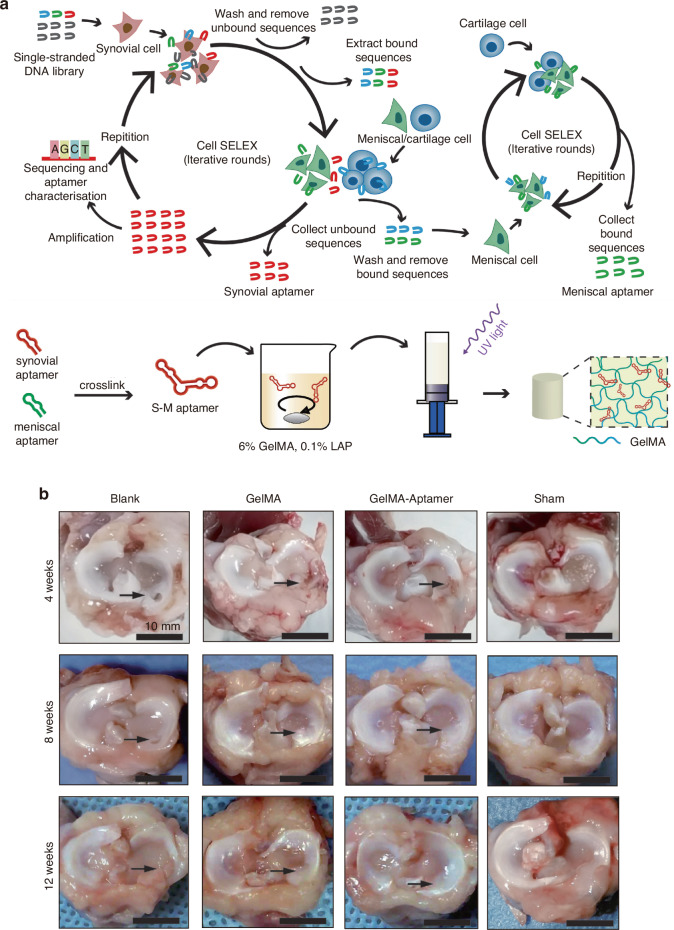
Preparation process of a bispecific GelMA-aptamer system and its therapeutic evaluation for meniscus defects. **a** Technological procedure for the filtration of aptamers and construction of the bispecific GelMA-aptamer system. **b** Observations of menisci at 4, 8, and 12 weeks post-surgery. Black arrows indicate the defect sites. Blank group, no repair; GelMA group, repair with GelMA hydrogel; GelMA-aptamer group, repair with GelMA hydrogel and bispecific aptamer; sham group, uninjured meniscus. Reproduced form ref. ^222^ with permission from SAGE Publications Inc., copyright 2023

In recent years, MSC transplantation and their directed differentiation into chondrocytes have become a preferred approach for cartilage repair.^214^ Nevertheless, exogenous stem cell transplantation frequently faces challenges in cartilage tissue engineering, such as the absence of a targeted delivery system for MSCs, the low survival rate of exogenous cells, and the risk of exogenous infection during in vitro culture and cell proliferation.^67,215,216^ Currently, an aptamer-bilayer scaffold for knee joint repair has been developed,^217^ comprising an aptamer-gel for cartilage regeneration and an aptamer-functionalized 3D graphene oxide-based biomineral framework for bone regeneration. MSC-specific aptamers anchored on this scaffold enable targeted MSC recognition, binding, and recruitment to osteochondral defect sites (Fig. 3a). The aptamer-gel incorporates kartogenin, a chondrogenic factor, which is released sustainably to accelerate MSC differentiation into chondrocytes for cartilage regeneration. As type II collagen abundance reflects regenerated cartilage quality and maturity,^218,219^ comparisons with the aptamer-bilayer scaffold reveal inferior type II collagen staining, reduced cell density, and limited subchondral bone formation in standalone aptamer-gel applications (Fig. 3b).^217^ This demonstrates the bilayer scaffold’s superior repair efficacy. Based on Apt19S, a DNA aptamer targeting pluripotent stem cells,^220^ Hao et al.^221^ proposed a meniscus regeneration strategy combining endogenous stem/progenitor cell (ESPC) recruitment with fibrocartilage formation. They developed a 3D scaffold featuring a biomimetic microstructure and the MSC-specific aptamer Apt19S. This scaffold can sequentially activate the homing of ESPCs and the differentiation of fibrocartilage, achieving superior meniscus regeneration and protection of articular cartilage. It presents a potential alternative for meniscus tissue regeneration and a ready-made cell-guided meniscus product for clinical applications. In addition, gelatin methacryloyl (GelMA) hydrogel loaded with circular bispecific synovial-meniscal aptamers has been proven to be effective for avascular meniscus repair by recruiting endogenous synovial and meniscus cells and facilitating fibrocartilage regeneration (Fig. 4a). In a rabbit meniscus defect model, this approach demonstrated fibrocartilage-like tissue formation, reduced cartilage degeneration, and improved mechanical strength 12 weeks post-operation (Fig. 4b).^222^ Aptamers’ role in articular cartilage and osteochondral therapy lies in their potential as novel biomaterials for repair and regeneration. Engineered aptamers can modulate post-injury inflammation by suppressing pro-inflammatory cytokines or accelerate repair by promoting fibroblast proliferation and differentiation. They may also serve as drug delivery components to enhance therapeutic precision and minimize side effects. However, translating these concepts into clinical practice requires further validation of aptamer safety, efficacy, and optimal application methods, including thorough investigation of in vivo biocompatibility, long-term stability, and tissue interactions. In summary, aptamers hold diverse therapeutic potential for cartilage repair, spanning inflammation regulation, tissue regeneration, and targeted drug delivery.

### Osteonosus

Osteonosus encompasses a broad spectrum of bone-related disorders involving diverse pathological mechanisms, including genetic, metabolic, inflammatory, or tumorigenic processes. These diseases may be congenital such as achondroplasia and osteogenesis imperfecta.^223,224^ Alternatively, these disorders may be acquired, such as diabetic bone disease, cancer-associated bone disorders, or osteoarthritis resulting from sports injuries.^225–227^ Similar to osteoporosis, osteonosus often involves genetic predisposition interacting with non-genetic factors, including endocrine imbalances, chronic inflammation, or systemic diseases. Under physiological conditions, bone tissue develops via intramembranous and endochondral ossification, initially forming weaker woven bone that is subsequently replaced by stronger lamellar bone.^228^ However, pathological states disrupt the balance between bone resorption and formation, leading to net bone loss and reduced bone mass.^229^ In summary, osteonosus represents a complex group of disorders frequently linked to genetic mutations, multifactorial pathogenesis, and impaired bone function.

#### Osteoporosis

In 1984, osteoporosis was defined as an age-related disease characterized by reduced bone mass and increased susceptibility to fractures.^230^ In 2001, the definition was revised to characterize osteoporosis as a skeletal disorder marked by compromised bone strength and an increased susceptibility to fractures, where bone strength is primarily determined by bone mineral density and bone quality.^231,232^ As the extent of population aging continues to deepen, osteoporosis emerges as an extremely crucial public health concern, imposing escalating socioeconomic burdens.^233^ In the USA, ~1.5 million fragility fractures occur annually,^234^ while UK epidemiology predicts lifetime osteoporotic fracture risks of 50% for women and 20% for men aged ≥50.^235^ Fracture risk escalates with age, particularly among the elderly, and is associated with substantial healthcare costs, functional impairment. The risk of hip fractures is particularly significant and is correlated with higher rates of disability and mortality.^231^

Osteoporosis pathogenesis centers on disrupted bone remodeling-resorption equilibrium. Estrogen potently inhibits osteoclast recruitment/activity in early menopause, suppressing bone resorption. Estrogen deficiency increases osteoclast numbers/lifespan while promoting osteoblast apoptosis, leading to trabecular thinning, cortical porosity, and accelerated remodeling, thereby causing bone mass loss.^236–238^ Remodeling initiates via osteoblast/osteocyte signals activating osteoclast-mediated resorption followed by bone formation.^231,239,240^ Excessive remodeling reduces bone mass and strength, promotes microdamage accumulation, and predicts fracture risk; lowering remodeling rates mitigates this risk.^231,241,242^ In osteoporosis, excessive remodeling degrades bone material properties, including bending resistance, elasticity, toughness, and strength.^231,243,244^ Moreover, osteoporosis is related to a decrease in the quantity and size of trabecular bones, their thinning, and transformation into a rod-like shape, replacing the stronger plate-like structures found in non-osteoporotic bone. In most osteoporosis patients, excessive remodeling may be the primary factor contributing to alterations in bone microarchitecture, as it leads to the loss of trabecular bone connectivity and further undermines the structural stability of the bone.^231,237^ In terms of mechanical loading, a deficiency in load will cause bone loss and an increase in remodeling activity.^245^ On the other hand, excessive loading can lead to an increase in microdamage.^246,247^ Microdamage is a reaction to repeated submaximal loads. It can sever lamellae and canaliculi, disrupt osteocytes communication, and induce osteocyte apoptosis. Subsequently, microdamage targeted for removal by osteoclasts is removed and repaired by osteoblasts.^231,248,249^ The optimal range of mechanical loading should fall between the two extremes, ensuring effective repair of microdamage without becoming excessive. Microdamage accumulates with advancing age, and it increases at a more rapid pace in women.^250^ In healthy postmenopausal women, bone remodeling doubles in the early stage and triples after 10–15 years.^251^ With increasing age, the body’s capacity to defend against oxidative stress diminishes. Some scholars have identified ROS as a contributing factor to the development of osteoporosis.^252,253^ Furthermore, the bone microstructure is disrupted to varying degrees, which impacts both bone quality and fracture resistance. Meanwhile, cortical bone undergoes structural changes because of aging and the menopausal transition.^254,255^ The occurrence of osteoporosis is also linked to genetic factors. Research has revealed that a single gene or a group of genes may determine bone mass at different skeletal locations. This suggests that genetic influences may affect an individual’s ability to accumulate and maintain bone mass throughout their lifetime, thus affecting the risk of developing osteoporosis.

A deficiency in estrogen increases the rate of bone remodeling. Even before menopause, a negative remodeling balance between bone resorption and formation can result in bone loss and compromise bone structure and strength^256^. Anti-osteoporosis medications reduce the rate of bone remodeling by either diminishing bone resorption or enhancing bone formation. For example, estrogen, selective estrogen receptor modulators, and bisphosphonates exert their effects primarily by inhibiting bone resorption, whereas intact parathyroid hormone functions as a powerful anabolic agent that increases bone formation.^257,258^ Currently, bisphosphonates are effective in reducing the risk of fractures and are relatively cost-effective. However, they are associated with gastrointestinal side effects. More critically, long-term application of bisphosphonates may simultaneously suppress the functions of both osteoblasts and osteoclasts. Even worse, prolonged administration may lead to osteonecrosis of the jaw and increase the risk of certain malignancies.^259,260^ In recent years, the exploration of various biological mechanisms in scientific research has deepened significantly. In the realm of osteoporosis research, an increasing number of studies indicate that the gut microbiota may serve as a potential target for the prevention and treatment of osteoporosis. Against this backdrop, the research concept of the “gut-bone axis” has gradually emerged and gained widespread attention. The nutrients ingested by the human body, along with the dietary patterns adopted, can influence the gut microbiota. In turn, changes in the gut microbiota may affect the host’s metabolic state, thereby regulating the process of bone metabolism. According to the gut-bone axis theory, dietary intake can participate in the regulatory mechanisms of bone metabolism by altering the abundance, diversity, and composition of the gut microbiota.^261–264^ An imbalance in the gut microbiota can result in intestinal inflammation, which releases various inflammatory factors such as TNF-α, IL-6, and IL-1β. These factors can further exacerbate the inflammatory response, causing adverse reactions such as damage to the intestinal mucosal barrier and immune dysfunction in the host, leading to the occurrence and progression of a series of diseases.^265–267^ These factors activate the RANKL signaling pathway, enhancing osteoclast function, which results in osteoporosis and bone loss.^268–270^ Nucleic acid aptamers can effectively modulate these inflammatory factors, alleviate inflammation, and subsequently influence bone metabolism.^271–275^ Multiple studies have shown that probiotics can enhance the integrity of the intestinal epithelium, prevent barrier degradation, and reduce pro-inflammatory responses.^276,277^ After effective probiotic intake in murines, there is an increase in the populations of bifidobacteria and lactobacilli in the gut, along with elevated levels of short-chain fatty acids (SCFAs), significant enhancements in bone density, and improvements in bone microstructure.^278–281^ SCFAs are key regulatory factors in osteoclast metabolism and bone mass.^282^ Propionate and butyrate can induce metabolic reprogramming in osteoclasts, leading to a downregulating of bone resorption and the protection of bone mass.^267^ Probiotic supplementation can significantly improve the gut environment and reduce the expression of inflammation-related factors.^283–286^ Research also indicates that a high-calcium diet combined with probiotic intervention can further amplify protective effects on murine bones, suggesting a potential synergistic relationship between diet and the microbiota. Probiotics can improve the efficiency of calcium absorption in the gut and enhance calcium utilization through the regulation of the gut microbiota, thereby increasing bone density and bone mineral content.^287^ For nucleic acid aptamers, their capacity to efficiently promote calcium absorption and foster the generation and survival of probiotics is a critical factor in determining their potential to improve the intestinal microenvironment. If nucleic acid aptamers demonstrate strong efficacy in these two areas, they could provide a novel and potent approach to optimizing gut microecology and boosting intestinal health.

Advancements in molecular biology and genetic engineering have promoted the development of aptamers, which serve as novel biomolecular tools that demonstrate significant potential in the study of osteoporosis. Furthermore, aptamers can regulate gene expression by specifically targeting nucleic acid sequences, thus affecting cellular functions related to bone formation and resorption. miRNAs represent a category of non-coding small RNAs that bind to target messenger RNAs (mRNAs). This binding results in either the degradation of the target mRNAs or the inhibition of their translation, ultimately regulating the expression of specific genes.^288–290^ Previous research has indicated a significant role of miRNAs in the process of bone remodeling, particularly in regulating the differentiation and function of both osteoblasts and osteoclasts.^291,292^ Furthermore, long non-coding RNAs are implicated in various signaling pathways and in the regulation of miRNAs during the process of bone formation.^293^ Three polymorphic loci within the FGF2 gene have been identified as being significantly correlated with femoral neck bone mineral density. Acting as potential binding sites for specific miRNAs, these loci may influence the expression of the FGF2 gene by altering the binding affinity between miRNAs and their target mRNAs.^294^ This suggests that modulating the activity of specific miRNAs could potentially serve as a novel therapeutic approach for osteoporosis. Circular RNAs (circRNAs), as a novel type of RNA molecule, have demonstrated significant importance in osteoporosis research. circRNAs influence bone metabolism by participating in the regulation of the differentiation, proliferation, and apoptosis of BMSCs.^295,296^ These research findings offer a new direction for the development of novel diagnostic markers and therapeutic targets. The previously mentioned CH6-LNPs-siRNA directly injects osteogenic Plekho1 siRNA into osteoblasts, leading to better bone microstructure and increased bone mass in tissues of osteopenic and healthy rats. Injecting an endothelial-specific aptamer-agomiR-195 system into aged murines can enhance the formation of CD31^hi^Emcn^hi^ blood vessels and reverse age-related osteoporosis (Fig. S1). Utilizing SELEX technology, endothelial-cell-specific aptamers are screened and aptamer candidate 2 (Table 1) with higher binding ability and a satisfactory secondary structure is selected. A small RNA molecule, agomiR-195, which mimics the function of miR-195, is synthesized. Subsequently, 1 volume of polyethyleneimine solution (100 μg/mL, pH 6.0) is mixed with six volumes of 4.2 μmol/L sodium citrate to form a polyethyleneimine-citric acid core structure (nanocore). Then, three volumes of the synthesized aptamers (50 nmol/L) and agomiR-195 (1 μmol/L) are added into the nanocore, and the mixture is reacted for 5 min to assemble into a nano-complex, namely aptamer-agomiR-195.^297^ Furthermore, an aptamer-antagomiR-188 targeting system has successfully and selectively silenced miR-188 in BMSCs, which in turn increases bone formation and reduces the accumulation of bone marrow fat in aged murines (Fig. S2).^298^ This system assembles into a nano-complex (Table 1) after obtaining the aptamer sequence with the highest binding affinity to murine BMSCs through the same technology previously described. Despite the significant potential of aptamers in osteoporosis research, several challenges persist regarding their clinical applications. Rapid renal filtration and metabolic instability result in the rapid clearance, degradation or structural alteration of aptamers, ultimately influencing their therapeutic efficacy. This occurs due to the relatively low molecular weight of aptamers, which enables them to easily traverse the glomerular filtration membrane, leading to a reduced half-life in the body and an inability to maintain adequate concentrations at the site of action, significantly impacting therapeutic efficacy.^299,300^ The glomerular filtration membrane has a specific pore size, typically around 4–5 nm, allowing small molecules to pass freely.^301^ The molecular weight of nucleic acid aptamers generally ranges from 5 to 15 kDa, which is considerably lower than the retention threshold of the glomerular filtration membrane (30–50 kDa), facilitating their smooth passage through the membrane and rapid clearance.^60,299^ Previous studies have chemically modified aptamers by conjugating them with macromolecules like polyethylene glycol (PEG) or cholesterol to enhance their molecular weight, which slows their passage through the glomerular filtration membrane and prolonging their half-life in the body.^302–304^ Furthermore, new drug delivery systems, such as nanoparticle carriers, can be developed to encapsulate aptamers, shielding them from rapid renal clearance and achieving targeted delivery, thus enhancing their stability and therapeutic effectiveness in the body. Additional issues such as polyanion effects, unexpected tissue accumulation, and non-specific immune activation further elevate the risk of potential side effects. Therefore, future studies must focus on strategies to enhance the biological properties and efficacy of aptamers while exploring effective methods to translate these findings into clinical therapeutic approaches.

#### Bone tumor

From a historical perspective, the classification of bone tumors has experienced numerous changes and amendments. As early as the 1950s, researchers had already initiated revisions to the classification of bone tumors; however, this classification still possessed certain limitations. Subsequently, additional research and findings have prompted further refinement of the classification. In 2013, the fourth edition of the World Health Organization (WHO) Classification of Soft Tissue and Bone Tumors updated the classification of bone tumors. The classification categorizes bone tumors into three categories based on their biological behavior: benign, intermediate (including subgroups of locally invasive and rarely metastatic), and malignant.^305^ Benign tumors only recur sporadically and are non-destructive. These tumors can be removed or curetted locally. The intermediate type is subdivided into locally invasive and rarely metastatic subtypes. Locally invasive tumors have a high recurrence propensity and show invasive and destructive growth, yet there is no risk of metastasis. Generally, extensive resection or the implementation of adjuvant measures is necessary for effective treatment. The rarely metastatic subgroup typically demonstrates locally invasive growth with a relatively low metastatic propensity of less than 2%. Malignant tumors are characterized by destructive growth, a high recurrence propensity, and a significant risk of metastasis, typically exceeding 20%. These tumors necessitate surgical intervention in conjunction with radiotherapy and/or chemotherapy.^306^ The most recent classification standard for bone tumors is the fifth edition released by the WHO in 2020. This edition does not introduce significant changes to the classification of bone tumors; however, it includes new tumor entities and provides descriptions of subtypes for existing tumor types. Meanwhile, it integrates new molecular and genetic data.^307^

##### Osteosarcoma

Osteosarcoma (OS) is a primary malignant skeletal tumor characterized by malignant mesenchymal cells that directly form immature bone or osteoid tissue.^308,309^ Typically, 80%–90% of osteosarcomas occur in long tubular bones. Conventional osteosarcoma accounts for ~15% of all primary bone tumors subjected to biopsy analysis. Among primary malignant bone tumors, its incidence is surpassed only by that of multiple myeloma.^309^ Patients are generally younger, with a higher prevalence in males than females, while those under 6 years old or over 60 years old rarely develop this disease.^309–311^ Its characteristics include a high propensity for metastasis, mainly to the lungs, and pathological fractures resulting from bone destruction.^312,313^ The etiology of osteosarcoma remains unclear, although evidence suggests that human osteosarcoma can be induced by viruses or cell-free extracts from human osteosarcoma, and ionizing radiation is a recognized environmental factor contributing to its development.^314^ Besides, osteosarcoma also shows genetic susceptibility, as several families have been reported with multiple members affected.^315^ Screening of a significant number of children with osteosarcoma reveals that around 3% to 4% carry germline mutations in the p53 gene, indicating a family history consistent with Li-Fraumeni syndrome.^316^

Through Cell-SELEX, the specific DNA aptamer OS-7.9 (Table 1) targeting MG-63 osteosarcoma cells has been successfully identified. It has been verified that the OS-7.9 aptamer has a greater affinity for osteosarcoma cancer cells, shows no affinity for human bone marrow neuroblastoma, and can bind to lung cancer and colorectal cancer cell lines.^317^ This suggests that there may be common markers shared between osteosarcoma cells and these cells, with the OS-7.9 aptamer potentially serving as a valuable probe in cancer research. It could be applicable in developing early diagnostic approaches and treatment methods for metastatic diseases, as well as in identifying common membrane proteins present in different cancer types. To improve the diagnostic capabilities for osteosarcoma, the selected single-stranded DNA aptamer LP-16 can specifically bind to osteosarcoma cells and is the first aptamer to identify metastatic osteosarcoma cells.^318^ Research has shown that combining aptamers with salinomycin or clustered regularly interspaced short palindromic repeat (CRISPR)-associated Cas9 nuclease (CRISPR/Cas9) can decrease tumor volume and malignancy in osteosarcoma.^319,320^

Metastatic bone tumor: Bone is a primary site for many tumor metastasis.^321^ The occurrence and metastasis of high-grade and high-stage cancers are associated with the heterogeneity deletion of specific gene loci.^322,323^ For instance, miR-15a and miR-16-1 are tumor suppressor genes located on chromosome 13q14 and are correlated with the expression of genes like BCL2, CCND1, and WNT3A, which play direct roles in cell survival, proliferation, and invasion processes.^324–328^ The loss of these genes may facilitate the survival, proliferation, and invasion of cancer cells. Aberrant expression of these miRNAs in tumor cells can lead to a more aggressive metastatic capacity. On the other hand, bone metastasis may also be influenced by interactions within the bone microenvironment. During the invasion of tumor cells, a variety of adhesion receptors are implicated. These adhesion events are of great significance for the invasion, metastasis, and ultimate establishment of new tumors by tumor cells. These adhesion receptors connect the extracellular environment with intracellular signaling by binding to extracellular ligands in the tumor microenvironment, thus enhancing tumor cell migration, invasion, proliferation, and survival.^329,330^ Tumor cells can colonize and proliferate within bone tissue through adhering to specific components. They are capable of stimulating the activity of osteoclasts, resulting in enhanced bone resorption. Conversely, they may also impact the function of osteoblasts and facilitate bone formation.^331^ This imbalance in bone metabolism creates a conducive microenvironment for tumor cells growth. Moreover, tumor cells secrete large amounts of pro-angiogenic factors, giving rise to an aberrant, disorganized, immature, and poorly permeable vascular network.^332^ The blood vessels supply oxygen and nutrients to tumor cells, and tumor angiogenesis remarkably accelerates growth and increases the metastatic potential of the tumor.^333^

Numerous aptamers have been shown to suppress tumor metastasis. For angiogenesis, Rana et al.^334^ demonstrated an aptamer-based dynamic platform for the spatiotemporal control of the bioavailability of an angiogenic growth factor by utilizing affinity interactions within a biofabrication-friendly polymer matrix, aiming to realize a mature and stable vascular network. The RNA aptamer A10-3.2 is a new ligand for bone-metastatic prostate tumors that expresses the prostate-specific membrane antigen and is conjugated with atelocollagen (ATE) to deliver miR-15a and miR-16-1. It synergistically induces selective death of prostate cancer cells and augments the anticancer efficacy of the system.^324^ As a specific aptamer targeting complement C5a, AON-D21 can block the C5a/C5aR1 signal axis, effectively reducing the bone metastasis and tumor burden in lung cancer (Fig. S3).^335^ Research indicates that BMSCs are capable of migrating from the primary tumor site to the bone marrow, with the migration dependent on osteopontin (OPN). When OPN is blocked by the R3 aptamer, BMSCs are unable to migrate to the bone marrow.^336^

##### Multiple myeloma

Multiple myeloma (MM) is a malignant plasma cell neoplasm characterized by clonal proliferation within the bone marrow microenvironment. Although it is classified as a hematologic malignancy, it exhibits a distinct propensity to develop almost exclusively within the bone marrow, leading to severe bone destruction. This occurs by augmenting bone resorption through osteoclasts while simultaneously suppressing bone formation.^337^ Consequently, MM is specifically addressed here for discussion and analysis.

The occurrence of bone disease in MM is analyzed at the cellular level. The predominant pathogenesis of MM-induced bone disease is attributed to the up-regulation of osteoclast differentiation and activity, resulting in unbalanced bone resorption and the initiation of characteristic lytic bone lesions.^338^ Several factors mediate the activation of osteoclasts in MM patients. MM cells directly secrete cytokines capable of generating osteoclasts, including IL-1, IL-3, IL-6, TNF-α, MIP-1α, MIP-1β, and decoy receptor 3.^339–344^ It has been previously mentioned that RANKL and OPG play a core role in osteoclast differentiation. The adhesion between MM cells and BMSCs triggers the expression of RANKL in osteoblasts and advances osteoclast differentiation by stimulating the NF-κB and Jun N-terminal kinase pathways. Therapeutic methods that normalize the RANKL/OPG ratio by increasing OPG and/or decreasing RANKL can effectively impede bone destruction and MM growth in vivo.^345^ Both MM cells and osteoclasts secrete elevated levels of the pro-inflammatory cytokine MIP-1α.^346^ MIP-1α facilitates the survival and migration of MM cells, stimulates the generation of osteoclasts by activating the ERK and AKT signaling pathways, and downregulates bone formation-related transcription factors (eg, osterix) to inhibit osteoblast differentiation, thereby reducing bone formation.^347–349^ Moreover, a recent study indicated that osteoclasts protect MM cells from T cell-mediated cytotoxicity by directly inhibiting proliferating T cells.^350^ This defective T cell-mediated reaction is a crucial mechanism for tumors to evade immune surveillance.^351^ The immunosuppressive function of osteoclasts and their key role in lytic bone diseases could be considered important targets for the treatment of MM.

The differentiation of MSCs into bone cells is governed by the activation of two principal transcription factors: runt-related transcription factor 2 (RUNX2) and osterix.^352^ These transcription factors are vital for the maturation and ossification of osteoblasts and depend on the typical Wnt signaling pathway.^353^ Whereas, myeloma cells secrete various Wnt antagonists like dickkopf-1 (DKK1) and sclerostin (SOST). These proteins compete for the same Wnt signaling receptors, suppressing the differentiation and function of osteoblasts.^354–358^ Studies conducted both in vitro and in vivo have demonstrated that blocking DKK1 and augmenting Wnt signaling can restore the quantity of osteoblasts and trabecular bone, along with a reduction in tumor burden.^359–362^ After treating myeloma murines in vivo with a SOST-neutralizing antibody, results indicated a decrease in bone loss and lytic lesions, although there was no impact on tumor burden.^363^

In terms of bone marrow adipocytes, while there is no direct connection with MM bone disease, emerging evidence shows that adipocytes support the growth and survival of MM, which is inversely related to bone mass.^353^ Adipocytes and osteoblast lineage commitment originate from common progenitor cells. Wnt signaling acts as the primary initiator for the osteoblast lineage while simultaneously suppressing adipocyte lineage commitment regulated by the PPARγ signaling pathway.^364,365^ As people age, bone marrow adipocytes increase, and the rate of bone formation declines.^353,366^ Obesity is positively correlated with the risk of MM occurrence and is associated with an increase in bone marrow lipid as well.^367–369^ Furthermore, bone marrow adipocytes are capable of secreting free fatty acids, which serve as an energy source for the proliferation of tumor cells.^370^ Many lipids function as ligands for nuclear receptors involved in the PPARγ signaling pathway.^371^ The levels of free fatty acids are generally elevated in MM patients.^372^ This may result in the up-regulation of the PPARγ signaling pathway, thereby enhancing bone marrow adipogenesis while inhibiting the differentiation of osteoblasts and supporting the survival of MM. A recent in vitro research has indicated that saturated fatty acids, specifically stearate and palmitate, exert a direct lipotoxic effect on human osteoblasts.^371,373^ Furthermore, the data illustrate that increased adipogenesis during obesity and aging undermines bone regeneration.^374^ In conclusion, bone marrow fat is emerging as a crucial regulatory factor in the development of MM and bone disorders, potentially representing a novel and promising therapeutic target.

**Fig. 5:**
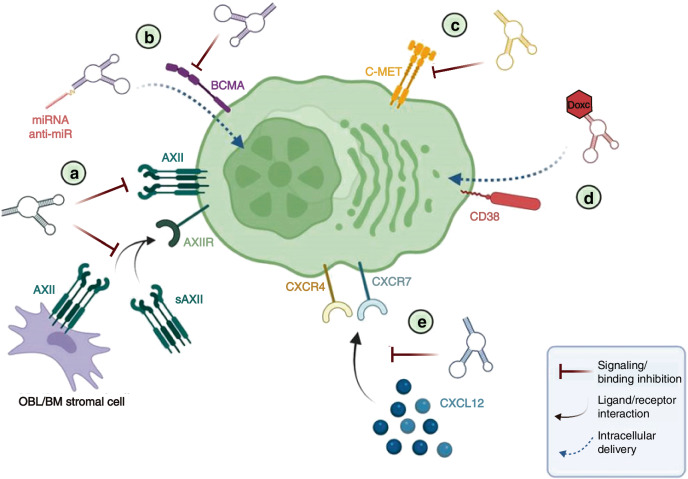
The potential application of nucleic acid aptamers in MM. **a** Aptamer against AXII. **b** BCMA targeted aptamer. **c** Aptamer against C-MET. **d** Conjugated CD38-doxorubicin aptamer and **e** NOX-A12 RNA aptamer for CXCL12. All the aptamers with the exception of the NOX-A12 spiegelmer are targeted at the receptors that are expressed on the plasma cell membrane of MM. BM bone marrow, Doxo doxorubicin, OBL osteoblast, sAXII soluble annexin A2. Reproduced form ref. ^387^ with permission from MDPI, copyright 2022

In the treatment of cancer, aptamers can serve as free molecules targeting specific cancer biomarkers as either agonists or antagonists (Fig. 5).^375^ Both AS1411 and NOX-A12, which have reached the clinical trial stage, are antagonistic aptamers that specifically target nucleolin and CXCL12, respectively.^376–379^ Nucleolin is highly expressed in multiple tumor cells. Through binding to the nucleolin receptor, AS1411 can play an anti-tumor effect.^380^ CXCL12 (also known as SDF-1) and CXC receptor 4 (CXCR4) are a chemokine and its receptor.^381^ CXCR4 is overexpressed in more than 75% of cancer cells, including those of myeloma.^382^ The CXCL12/CXCR4 pathway is a known crucial regulatory factor for tumor proliferation, cell spread, and migration both within and outside the bone marrow.^383,384^ NOX-A12 has been evaluated in research studies when used in combination with bortezomib and dexamethasone for treating relapsed MM.^377^ It produces a therapeutic effect on MM by preventing the interaction between CXCL12 and its receptor, thereby interfering with the CXCL12/CXCR4 signaling pathway and inhibiting the growth and proliferation of MM cells. What’s more, the specific aptamer ola-PEG for stromal cell-derived factor-1 (SDF-1) can neutralize SDF-1 and block SDF-1-dependent signal transduction, effectively suppressing the progression of MM.^385^

Annexin A2 (AXII) is overexpressed in the plasma cell membrane of MM, and its expression correlates negatively with patient survival rates.^386^ AXII, a member of the annexin family, is calcium-dependent and binds to phospholipids. The interaction between AXII and its receptor AXIIR augments the adhesion and growth of MM cells within the bone marrow microenvironment,^387^ potentially supporting the homing and growth of MM cells in this niche. AXII can be secreted by diverse cell types within the bone marrow, facilitating the growth of MM cells by establishing a pro-tumorigenic niche. Therefore, targeting the AXII/AXIIR axis represents a promising strategy for developing treatments against the MM niche.^388^ Zhou et al.^389^ have identified an ssDNA aptamer (wh6) that can specifically bind to MM cells expressing AXII both in vitro and vivo, and it can also suppress the adhesion and progression of MM cell lines induced by AXII. This shows that the wh6 aptamer is suitable for targeted MM therapy.

B cell maturation antigen (BCMA), a member of the TNF receptor superfamily, is preferentially expressed by mature B lymphocytes but is rarely expressed in hematopoietic stem cells.^390^ The ligands for BCMA, such as B-cell activating factor and a proliferation-inducing ligand (APRIL), can activate the NF-κB pathway.^387,391,392^ Nevertheless, the overexpression of BCMA and increased activation correlate with the progression of MM. These key genes exhibit a state of overexpression, facilitating the upregulation of the NF-κB pathway as well as the growth and survival of MM.^390,393^ In this context, Catuogno et al.^393^ selected a BCMA-targeted internalizing RNA aptamer (apt69.T) via Cell-SELEX, which demonstrated the inhibitory capacity on the APRIL-induced NF-κB pathway.

Hepatocyte growth factor (HGF) constitutes a specific ligand for the tyrosine kinase receptor C-MET.^394^ In MM, the expression of C-MET progressively augments during disease progression, and its elevated expression is correlated with a poorer prognosis for MM patients. Upon binding of HGF, C-MET dimerizes and initiates the processes that promote cell growth, migration and angiogenesis while inhibiting apoptosis, ultimately enhancing the development of MM.^395^ SL1 is the truncated form of the original CLN0003 ssDNA aptamer. This aptamer was obtained via the filtered SELEX method against purified C-MET to identify tumors with overexpressed C-MET.^396,397^ Previous research has proven that targeting C-MET with the SL1 aptamer can inhibit HGF-dependent C-MET signaling and restrain the growth of MM cells.

CD38 is a kind of cell surface glycoprotein that shows especially extensive and high expression levels in MM, contributing to the disease’s progression.^387,398^ Consequently, it has become one of the primary targets for developing anti-MM targeted therapy in recent years. Wen et al.^399^ identified a CD38-specific ssDNA aptamer using a hybridization protein and Cell-SELEX method. Subsequently, doxorubicin was non-covalently coupled to create a CD38-specific aptamer drug conjugate. It can be readily internalized by MM cells. Following pH-dependent release in lysosomes, doxorubicin is capable of exerting its specific anti-tumor activity by inhibiting tumor growth in both in vitro and vivo models.^387^

#### Osteoarthritis

**Fig. 6:**
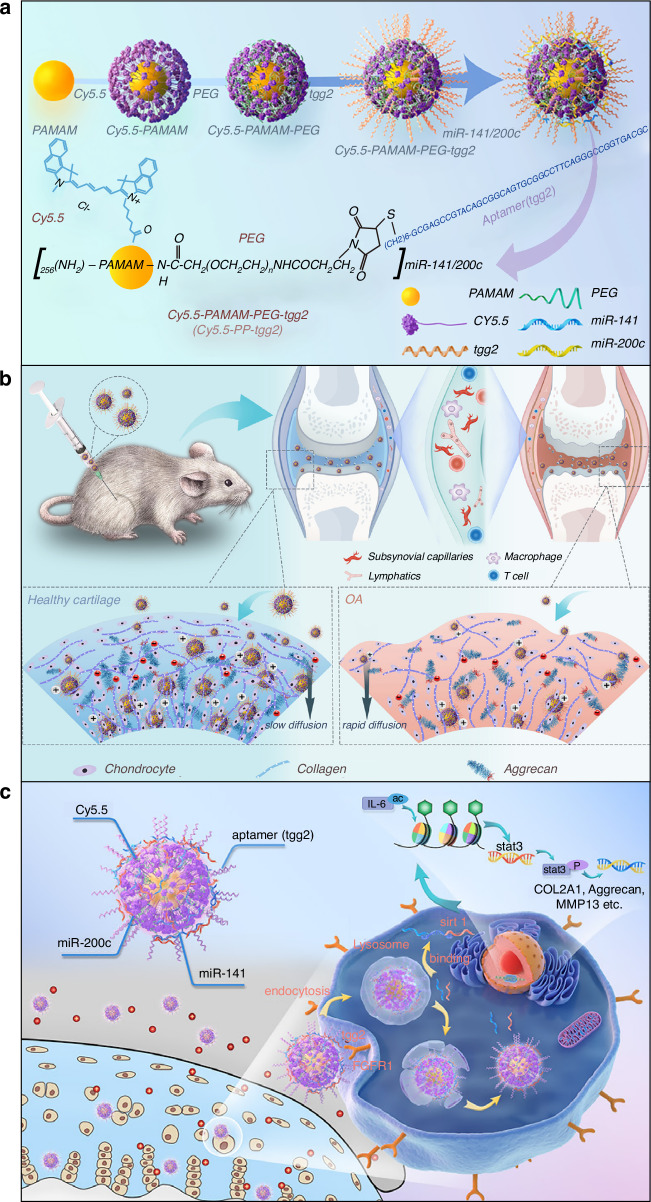
The mechanism of nucleic acid aptamers on chondrocytes in the pathological process of OA. **a** Schematic representation of the synthesis of tgg2-PEG2000-PAMAM6.0-Cy5.5. **b** Differentiated diffusion models of cationic nanocarriers binding to the negatively charged extracellular matrix (ECM) of chondrocytes via electrostatic interactions, followed by delivery into chondrocytes in normal and OA cartilage. **c** Modulation of the SIRT1/IL-6/STAT3 signaling pathway by miR-141/200c. Reproduced form ref. ^410^ with permission from Elsevier, copyright 2021

Osteoarthritis (OA) is a degenerative joint disease characterized by joint pain, tenderness and restricted mobility. As the most prevalent form of arthritis globally, it exhibits the highest incidence among all arthritis types.^400^ Evidence suggests that ~240 million individuals worldwide suffer from symptomatic OA, making it a leading contributor to physical disability and diminished quality of life.^401,402^ The primary therapeutic goals are pain management and avoidance of treatment-related toxicity.^403^ The knee joint is the most frequently affected site, with higher prevalence among the elderly, particularly females.^400,404–406^ Originally, OA was attributed to mechanical wear of articular surfaces;^407^ however, it is now recognized to involve genetic predisposition, aging, hormonal influences, lifestyle factors, and other contributors. Its typical pathological characteristics include degeneration of articular cartilage, sclerosis of subchondral bone, synovial lesions, and joint inflammation.^408,409^ In the synovial joint of the knee, critical structures such as the meniscus, articular cartilage, subchondral bone, and synovium collectively mediate these pathological changes while maintaining joint functionality. Ji et al.^410^ revealed significant dysregulation of miR-141/200c in OA cartilage tissue. Regulating miR-141/200c levels profoundly affects chondrocyte proliferation, apoptosis, and metabolic markers. Utilizing a Cell-SELEX system, researchers identified chondrocyte-specific aptamers (tgg2, tgg5, tgg8) (Fig. S4). The tgg2 aptamer possesses a more satisfactory secondary structure and shorter nucleotide sequence for nanoparticle conjugation. Based on this, an aptamer (tgg2)-PEG2000-PAMAM6.0-cy5.5 nanoplatform for delivering miR-141/200c was developed (Fig. 6a). In the destabilized medial meniscus model of OA, intra-articular injections of miR-141/200c inhibitors significantly alleviate the OA pathology via SIRT1/IL-6/STAT3 pathway modulation, reduces chondrocyte apoptosis, and increases pain thresholds (Fig. 6b, c).

**Fig. 7:**
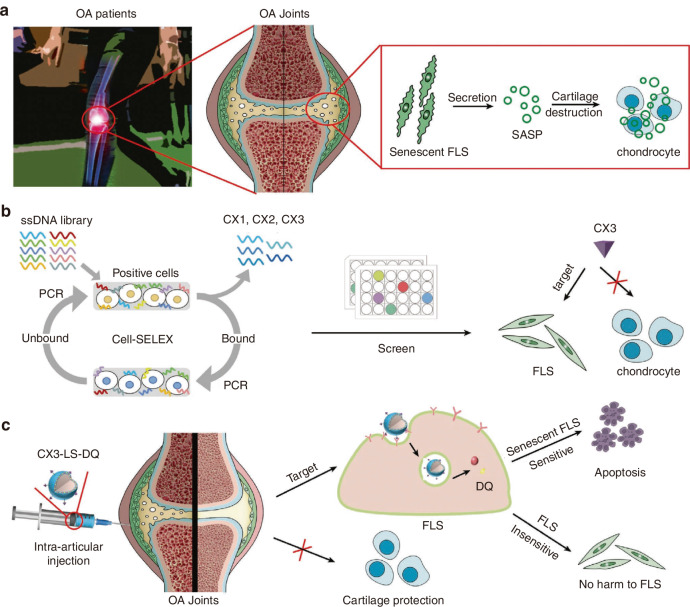
Schematic diagram illustrating the pathogenesis of OA and the effects of targeted eliminating of senescent FLS on the progression of OA. **a** FLS were predominantly senescent during the progression of OA, promoting chondrocyte dysfunction. **b** FLS-targeted aptamers were screened using Cell-SELEX system, and aptamer CX3 was identified as a specific targeting ligand for FLS, not chondrocytes. **c** Intra-articular injection of CX3-modified liposomes (CX3-LS) loaded with senolytics could effectively induce the apoptosis of senescent FLS without affecting the cartilage, thereby suppressing cartilage degradation. Reproduced form ref. ^420^ with permission from Wiley-VCH Verlag GmbH, copyright 2022

The synovium is composed of fibroblasts and macrophages. It produces synovial fluid containing lubricants and hyaluronic acid, which lubricates the joints and nourishes the articular cartilage.^411–415^ Synovitis is characterized by the proliferation of fibroblast-like synoviocytes (FLS) and the recruitment of macrophages, resulting in the hyperplasia of the synovial lining.^416^ Among the numerous factors that activate FLS in OA, follistatin-like protein 1 (FSTL1) is overexpressed in the synovium of OA patients, and its level correlates with OA severity.^417^ Activated FLS in OA secrete pro-inflammatory cytokines, chemokines, and proteolytic enzymes, contributing to inflammation propagation and cartilage matrix degradation.^418^ These responses and elevated production of inflammatory cytokines give rise to leukocyte recruitment, macrophage accumulation, and subsequent osteoclast activity enhancement.^419^ Primary FLS isolated from OA patients for in vitro experiments confirmed that most OA-FLS are senescent and capable of inducing chondrocyte apoptosis and an OA-like phenotype.^420^ As people age, the accumulation of senescent cells in the body has been identified as a contributing factor to OA.^421^ Chen et al.^420^ utilized Cell-SELEX technology to screen aptamers (CX1, CX2, and CX3) targeting FLS, with CX3 showing superior binding specificity. Consequently, CX3-conjugated liposomes (LS) were developed to eliminate senescent FLS, reducing non-specific toxicity and side effects while providing a novel therapeutic strategy for OA (Fig. 7).

Furthermore, inflammatory factors play a pivotal role in the pathological process of arthritis. In a specific study, the function of inflammatory factors in post-traumatic OA was examined using an “idealized“ anterior cruciate ligament reconstruction (IACL-R) model. The research discovered that although the IACL-R surgery restored normal gait characteristics, the concentrations of all tested inflammatory factors, including IL-1α, IL-1β, IL-2, IL-6, IL-8, IL-18, TNF-α, and granulocyte-macrophage colony-stimulating factor, in synovial fluid significantly increased on days 3, 7, and 15 following the surgery.^422^ These factors can activate multiple cell types within the joint, including fibroblasts, macrophages, and T cells, initiating a cascade of inflammatory reactions.^423^ Inflammatory cytokines are crucial in the pathogenesis of OA, significantly contributing to the disruption of metabolic homeostasis within joint tissues by promoting catabolism and destruction. In the pathogenesis of OA, these cytokines influence the production of other cytokines, inflammatory compounds and enzymes through combinatorial intracellular signaling pathways.^423^

IL-1 is a crucial cytokine involved in the pathogenesis of OA, capable of inducing inflammatory responses and catabolic effects.^423^ IL-1 inhibits chondrocytes from synthesizing ECM components and disrupts the synthesis of key structural proteins, such as type II collagen and aggrecan.^424–426^ Furthermore, it facilitates the production of two ECM-degrading enzymes: a disintegrin and metalloproteinase with thrombospondin motifs (ADAMTS) and matrix metalloproteinases (MMPs). It induces chondrocyte apoptosis and stimulates the production of other cytokines in an autocrine manner, thereby promoting hydrolysis.^427–430^ IL-1α, a member of the IL-1 family, is a cytokine that exerts a crucial function in the modulation of immune responses and participates in various diseases. Ren et al.^272^ obtained the aptamer SL1067 (Table 1), which exhibits high affinity and remarkable specificity for human IL-1α through cell-SELEX screening. Comprised of only 22 nucleotides, it achieves high-affinity binding, representing the smallest modified aptamer to be crystallized, and acts as an effective inhibitor of the IL-1α/IL-1 receptor pathway signaling. IL-18 is another representative of the IL-1 family.^431^ Research has revealed that the levels of Caspase 1 in the articular cartilage and synovium of patients with OA are elevated, significantly promoting the formation of IL-1β and IL-18.^432^ The production of IL-18 in joints is determined by chondrocytes, osteoblasts, FLS and macrophages.^433–435^ The level of IL-18 is elevated in the articular cartilage and synovium of OA patients and is positively correlated with disease severity.^436–438^ IL-18 can induce the upregulation of IL-18 receptors on chondrocyte surfaces and stimulate the synthesis of MMP-1, MMP-3, and MMP-13.^439^ In addition to increasing cartilage-degrading enzyme concentrations, it suppresses the production of proteoglycans, aggrecan, and type II collagen. Furthermore, chondrocytes exhibit typical apoptotic morphological changes.^440–442^ It facilitates the generation of multiple compounds and enzymes by chondrocytes and synoviocytes in an autocrine fashion.^433,443–446^

TNF-α and IL-1 are collectively recognized as crucial inflammatory cytokines involved in the pathophysiological process of OA. They hinder the synthesis of proteoglycan components, protein-bound proteoglycans, and type II collagen in chondrocytes.^447,448^ TNF-α exerts effects similar to those of IL-1β. During the progression of OA, there is a distinct synergistic interaction between these two cytokines.^449^ They promote MMP and ADAMTS-4 production, reduce the efficiency of the mitochondrial respiratory chain, and induce chondrocyte death and aberrant progenitor cell migration.^430,450–453^ Furthermore, chondrocytes influenced by IL-1β and TNF-α exhibit accelerated senescence and apoptosis.^451,452,454^ Additionally, TNF-α enhances the synthesis of IL-6, IL-8, regulated upon activation normal T cell expressed and secreted factor, and VEGF.^455–458^ Using SELEX technology, Orava et al.^273^ screened a DNA aptamer with a specific variable region (VR) sequence that binds to TNF-α. Among these, VR11 (Table 1) is the sole aptamer capable of inhibiting human TNF-α function without inducing the innate immune response associated with the production of inflammatory cytokines.^273^

IL-6 is a cytokine with multifaceted interactions, capable of activating the immune system and intensifying the inflammatory response.^423^ Its primary role involves promoting the formation of osteoclasts, thereby facilitating bone resorption, and it exhibits synergistic effects with IL-1β and TNF.^459,460^ Polymorphisms in its gene may predict the development of OA.^461,462^ IL-6 acts in concert with other factors to decrease type II collagen production and increase MMP synthesis.^463–465^ Furthermore, experiments on IL-6-deficient murines have demonstrated that, compared with wild-type murines, they tend to present more severe degenerative alterations.^466^ Gupta et al.^271^ isolated IL-6 SOMAmers through the SELEX process, which possess high affinity, slow complex dissociation, nuclease resistance, and effectively inhibit IL-6 signaling.

IL-8 belongs to the C-X-C chemokine family and predominantly exhibits chemotactic activity towards neutrophils. Although these cells typically do not produce high levels of IL-8 under quiescent conditions, they generate elevated concentrations upon agonist stimulation. Nevertheless, IL-8 is highly expressed in some tumor cells.^467^ IL-1 and TNF are the most potent inducers of IL-8, while other cytokines such as IL-6 do not effectively signal its production. The expression of IL-8 is cell-specific and stimulus-specific.^468,469^ Sung et al.^274^ have successfully developed and characterized the 20-fluoro-pyrimidine-modified RNA aptamer 8A-35 (Table 1), which can recognize human IL-8. This aptamer demonstrates highly specificity/affinity and effectively neutralizes human IL-8. The 8A-35 aptamer modulates various biological activities of IL-8 in human neutrophils, including intracellular signal transduction and chemotaxis.

IL-17 represents a family of cytokines with inflammatory functions. Its primary sources include stimulated CD4 ^+^ T cells and mast cells, which infiltrate the synovial membrane and the entire joint via blood vessels.^470–472^ Several studies have demonstrated that these T cells mediate direct immune response against chondrocyte and fibroblast membrane antigens during OA.^473^ IL-17 has been demonstrated to suppress proteoglycan synthesis in chondrocytes and promote the production of MMPs.^474–476^ It also secretes other cytokines and compounds that negatively affect cartilage.^477–479^ Besides, IL-17 facilitates VEGF secretion, contributing to synovial hypertrophy.^458,480^ IL-17 gene polymorphisms may correlate with OA susceptibility.^481^ As a member of the IL-17 family, IL-17A is associated with a variety of autoimmune diseases. Ishiguro et al.^275^ screened aptamers Apt3 and Apt21 and obtained optimized short aptamer derivatives (Apt3-4 and Apt21-2) through chemical and functional manipulation. In normal human dermal fibroblasts, Apt21-2 significantly blocks IL-17A-induced phosphorylation of signaling factors and IL-6 expression, suppressing inflammatory lesions and neurological symptoms, thus demonstrating therapeutic potential for autoimmune diseases. Adachi et al.^482^ prepared the RNA aptamer AptAF42dope1, which exhibits a higher affinity for IL-17A/F, offering novel biotechnological and medical applications.

IL-15 is one of the crucial cytokines in rheumatoid arthritis pathogenesis.^483,484^ Its function primarily involves stimulating the differentiation and proliferation of T cells and natural killer cells.^485^ During the pathogenesis of OA, elevated IL-15 concentrations in synovial fluid at early disease stages stimulate secretion of specific MMP-group metalloproteinases.^486^

#### Osteogenesis imperfecta

Osteogenesis imperfecta (OI) is a hereditary connective tissue disorder primarily characterized by fragile bones.^487^ The etiology of OI is mainly linked to genetic mutations, particularly those involving the COL1A1 or COL1A2 genes, which encode the alpha-1 and alpha-2 chains of type I collagen, a key component of the skeletal structure. Abnormal synthesis of these proteins impacts bone strength and architecture.^488,489^ The clinical manifestations of OI are highly heterogeneous. Depending on the severity and type, patients may present symptoms such as dentinogenesis imperfecta, blue sclera, short stature, hearing loss and cardiac anomalies.^487,490^ OI is typically categorized into four main types: Type I (the most common and mild form), Type II (associated with neonatal mortality), Type III (severe), and Type IV (moderate to severe).^491^ The incidence of OI is ~1 in 15 000 to 20 000 live births, with no gender, racial, or ethnic predilection.^488,492,493^ The primary treatment goals for OI are to prevent fractures, manage symptoms, and increase bone mass; however, currently available therapeutic options have limited efficacy.^494,495^ Commonly used medications, such as bisphosphonates, denosumab, synthetic parathyroid hormone, and growth hormone for children, can improve bone density and reduce fracture incidence to some extent. Nonetheless, their effectiveness remains suboptimal, and they may be ineffective in certain patients or induce cytotoxic side effects.^494^

Sclerostin, a glycoprotein secreted by mature osteocytes, antagonizes the Wnt/β-catenin signaling pathway by binding to low-density lipoprotein receptor-related proteins 5 and 6 (Lrp 5/6), thereby inhibiting bone formation.^496–499^ In clinical trials targeting low bone mass disorders such as osteoporosis and OI, sclerostin antibody (Scl-Ab) therapy has been shown to interfere with sclerostin, consequently increasing bone formation and mass.^500,501^ Clinically, the humanized therapeutic Scl-Ab (romosozumab) has demonstrated bone anabolic potential in postmenopausal osteoporosis, though serious cardiovascular adverse events have been reported.^502–504^ Given that cardiovascular abnormalities are a secondary feature in OI patients, Scl-Ab treatment may elevate cardiovascular risks, particularly in those with preexisting cardiovascular conditions.^487,505,506^ Ge Zhang et al.^507^ developed next-generation sclerostin inhibitors to promote bone formation without exacerbating cardiovascular risks in OI. The study designed multiple animal models. The *Col1a2*^*+/G610C*^ murine model was established for OI investigation. Another model, *hSOST*^*ki*^:*Col1a2*^*+/G610C*^, was generated by knocking in human sclerostin (hSOST) into *Col1a2*^*+/G610C*^. The *Δloop3-hSOST*^*ki*^:*Col1a2*^*+/G610C*^ model was further modified from *hSOST*^*ki*^:*Col1a2*^*+/G610C*^, where *Δloop3* denotes the deletion of the loop3 domain of sclerostin. This model was utilized to examine the role of the loop3 domain of sclerostin in bone formation and the cardiovascular system. Additionally, an aortic aneurysm (AA) and atherosclerosis murine model, *ApoE*^*−/−*^, was constructed based on the previous three models.

In vitro primary osteoblast experiments demonstrated that overexpression of full-length sclerostin (FL hSOST) significantly inhibited the Wnt signaling pathway and downregulated osteogenic markers, confirming the sclerostin’s anti-anabolic role. In contrast, cells overexpressing loop2 and/or loop3 deficient sclerostin exhibited increased Wnt signaling and osteogenic marker expression, underscoring loop3’s critical role in sclerostin-mediated bone inhibition (Fig. S5a). Micro-CT and bone histomorphometric analysis revealed that sclerostin’s loop3 domain is pivotal in bone regulation. Compared to *Col1a2*^*+/G610C*^ murines, *hSOST*^*ki*^:*Col1a2*^*+/G610C*^ murines (expressing FL hSOST) displayed significantly poorer trabecular bone volume and microstructure. In contrast, *Δloop3-hSOST*^*ki*^:*Col1a2*^*+/G610C*^ murines demonstrated notable improvements in these bone parameters relative to the *hSOST*^*ki*^:*Col1a2*^*+/G610C*^ murine model, indicating that loop3 deletion mitigates sclerostin’s inhibitory effects (Fig. S5b, c).

The construction of AA and atherosclerosis models and measurement of relevant indicators revealed that, compared to *Col1a2*^*+/G610C*^:*ApoE*^*−/−*^, both *hSOST*^*ki*^:*Col1a2*^*+/G610C*^:*ApoE*^*−/−*^ and *Δloop3-hSOST*^*ki*^:*Col1a2*^*+/G610C*^: *ApoE*^*−/−*^ exhibited significantly reduced AA incidence, smaller maximum diameters of the aortic arch and renal artery, lower proportions of atherosclerotic lesions at the aortic root, and decreased serum levels of IL-6, TNFα and monocyte chemoattractant protein-1 (MCP-1) (Fig. S6). No significant differences in these cardiovascular-related parameters were observed between the *hSOST*^*ki*^:*Col1a2*^*+/G610C*^:*ApoE*^*−/−*^ and *Δloop3-hSOST*^*ki*^:*Col1a2*^*+/G610C*^:*ApoE*^*-/*^ groups. These findings indicate that sclerostin lacking the loop3 domain confers a cardiovascular protective effect similar to FL hSOST, suggesting that sclerostin’s cardiovascular protection is independent of the loop3 domain. Apc001PE, a PEG40k-conjugated modified aptamer targeting the loop3 domain of sclerostin (Table 1), was developed based on its precursor aptscl56. Aptscl56 binds specifically to the loop3 domain of sclerostin in the serum of OI patients, inhibiting sclerostin’s antagonistic effects on the Wnt signaling pathway and osteogenic potential. Notably, this inhibition does not interfere with sclerostin’s suppressive effects on inflammatory cytokines/chemokines or elevate cardiovascular risk. Furthermore, Apc001PE demonstrated no toxicity in healthy murine and rat models, highlighting its potential as a novel therapeutic agent with significant clinical implications for OI treatment.

In subsequent research, Ge Zhang’s team further addressed the issue of lower affinity in the aptamer Aptscl56.^508^ They introduced seven distinct quinoline derivatives (spanning 2-quinoline to 8-quinoline) at key sites of Aptscl56 to synthesize a modified aptamer library. The binding ability of these derivatives to sclerostin was evaluated against the unmodified Aptscl56, revealing that the 5-quinoline-modified aptamers enhanced binding ability by 6 to 16 times at multiple critical sites, outperformed other modifications, and exhibited a lower dissociation constant. Following enzyme-resistant modification, the 5-quinoline-modified aptamer demonstrated further improved binding efficacy. In vitro cell experiments showed that the 5-quinoline-modified aptamer more robustly restored Wnt signaling pathway activity and bone formation marker expression. In vivo animal experiments confirmed its pronounced bone-forming effects in murine models compared to the unmodified aptamer. Molecular dynamics simulations and experimental analyses revealed that the 5-quinoline modification altered the aptamer-sclerostin binding conformation, diverging significantly from the natural Aptscl56 interaction.

## The other applications of nucleic acid aptamers

Nucleic acid aptamers not only demonstrate significant potential in treating orthopedic diseases but also exhibit broad applications across biomedical fields. These applications highlight the multifunctionality of aptamer technology and provide novel approaches for disease research and therapy. For instance, advancements in aptamer-based biosensing enable precise diagnostic methods, offering powerful tools for early disease detection and progression monitoring. Biological detection utilizes biological principles and techniques to detect, quantify, or monitor various substances, biological processes, or environmental conditions, through analysis of cells, proteins, nucleic acids, and metabolites. This technology is critical for early health threat detection, drug safety/efficacy evaluation, bioequivalence assessment, and dosing regimen optimization.^509,510^ With Next-Generation Sequencing (NGS) technology, it plays an increasingly important role in diagnosing genetic diseases like cancer.^511^ However, population-wide implementation remains limited, necessitating new biomarkers to bridge gaps between exposure and disease onset. Success in biological detection hinges on data quality and actionable insights from analyses. As personalized medicine advances, rapid genomic testing becomes increasingly vital. Overall, biological detection is a multidisciplinary field essential to public health, environmental protection, drug development, and personalized medicine. Detection technologies such as fluorescence, electrochemical, and nucleic-acid-aptamer-based biosensors have gradually matured.^512–516^ Nucleic-acid-aptamer-based detection technology relies on the high affinity and specific binding between aptamers and specific target molecules. SELEX-derived aptamers recognize specific molecules and, when integrated with other technologies, generate quantifiable signals.^512,513,515,516^ Their unique 3D structures ensure specificity and sensitivity, while in vitro synthesis enables scalable production and functional modifications.^512,515^ Compared to antibodies, aptamers exhibit superior stability and environmental adaptability.^515^ Aptamer biosensors offer the advantages of rapid response, simple operation, and low cost.^512,513,516^ Moreover, they have been successfully applied in the detection of various substances, demonstrating promising application prospects.^513,515^ Furthermore, in further research, efficient signal transduction can be readily achieved, enabling sensitive and selective detection as well as high-throughput drug screening.^515^ Aptamers possess extensive target-binding capabilities and can be utilized to construct sensors for multi-substance detection.^514,516^ They demonstrate significant potential in disease diagnosis, food safety detection and environmental monitoring.^517–526^

**Fig. 8:**
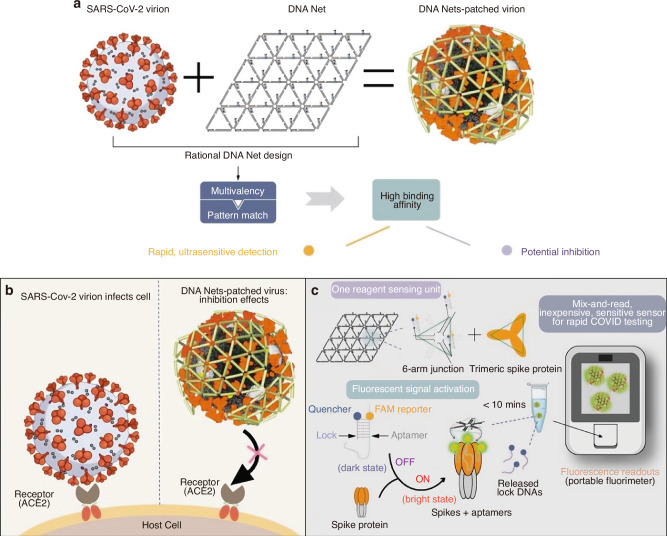
The potential of nucleic acid aptamers in biological detection. **a** The technological procedure and construction of the DNA Net-aptamers. **b** The complex of DNA Net-aptamers are attached to a virion can block the interactions between the spike protein and ACE2 receptors on the surface of a host cell, inhibiting viral infection due to its high neutralization potency. **c** The principle of signal output for DNA Net aptamers. Reproduced form ref. ^527^ with permission from American Chemical Society, copyright 2023

During the SARS-CoV-2 pandemic, early nucleic acid and antigen tests faced limitations including reagent shortages, complex procedures, and high costs.^527,528^ Polymerase chain reaction (PCR) could not confirm viral infectivity, and antigen detection lacked sensitivity.^529^ To address this, a macromolecular DNA nanoscaffold (DNA Net) was designed based on the trimeric spike protein structure of SARS-CoV-2 (Fig. 8a). A tri-aptamer targeting the spike’s receptor-binding domain (RBD) enabled rapid, sensitive virus detection and inhibition (Fig. 8b).^27,527^ Moreover, an aptamer switch was designed to transform DNA Net-aptamers into a highly sensitive biosensor for virus detection via fluorescence readings (Fig. 8c). Subsequently, molecular docking and dynamics simulations validated the design, while SPR confirmed that DNA Net aptamers (especially larger constructs) enhanced binding affinity.^527^ Chauhan et al.^527^ identified the optimal aptamer-lock candidate pair (Apta-lock-1) and utilized a 4 × 4 DNA Net for their research, demonstrating that multivalent aptamer-spike interactions improve SARS-CoV-2 detection sensitivity. This system detects viruses without nucleic acid extraction and amplification, fulfills early-infection sensitivity requirements, and differentiates infectious from inactivated viruses, reducing false positives. Increased DNA scaffold flexibility can enhance virus detection sensitivity. More importantly, it exhibits selectivity, with no cross-reaction against H1N1, OC43 and Zika virus, and it can detect SARS-CoV-2 variants, albeit with relatively lower signal intensity and sensitivity for these variants. DNA Net aptamers also show therapeutic potential, inhibiting SARS-CoV-2 in vitro with nearly 1 000-fold greater efficacy than monomeric aptamers.

## Conclusion and perspective

Nucleic acid aptamers, as short oligonucleotide sequences, form specific secondary/tertiary structures enabling high-affinity target binding. Ranging from traditional SELEX technologies to novel techniques such as Pro-SELEX and microfluidic-chip-SELEX, screening techniques continue to evolve, improving efficiency and specificity. Furthermore, aptamer-target interactions involve conformational changes and multimodal binding, with tunable affinity. Regarding applications, nucleic acid aptamers have demonstrated substantial potential across various fields, including biosensors, repair, oncology, and therapy.

Despite significant advancements in the field of nucleic acid aptamers, several areas warrant further investigation and exploration. Research on DNA, siRNA, and miRNA within the scope of nucleic acid aptamers has been relatively comprehensive and well-documented. However, research concerning circRNA remains somewhat limited. Given that RNA aptamers and RNA-aptamer-based devices are constrained by low expression levels and rapid degradation in mammalian cells.^530^ circRNAs are characterized by a covalently closed loop structure resulting from a specialized alternative splicing process known as back-splicing, where the originally linear mRNA molecules form loops.^531–534^ This unique structure endows circRNAs with diverse roles within organisms, such as acting as miRNA sponges, serving as protein scaffolds, and functioning as translation templates. While it is known that they play roles in various diseases, many mysteries remain concerning the specific functions of most circRNAs and their mechanisms of gene expression regulation.^535^ The complexity of circRNA research is also reflected in their expression specificity; circRNAs exhibit both cell-type and tissue-specific expression, which can be largely independent of their corresponding linear host genes' expression levels.^536^ Even within the same species, across different tissues or cell types, certain circRNAs may exhibit divergent expression patterns, further complicating the research. Furthermore, although some circRNAs contain start codons, experimental results indicate that circRNAs with AUG sequences cannot bind to ribosomes and thus are not translated.^537^ However, whether all circRNAs are incapable of participating in protein translation remains currently under investigation, suggesting they may not function through the conventional translational pathway. The biosynthetic pathways of circRNAs also present a research challenge. Their synthesis is complex and exhibits low efficiency. While chemical cyclization methods can facilitate the synthesis of circRNA, they involve strict reaction conditions, low yields, and a propensity to generate by-products, such as 2'-5' phosphodiester bonds, which restrict their application in nucleic acid aptamers.^538,539^ Enzymatic strategies can enhance cyclization efficiency but necessitate specific enzymes and complex reaction conditions, while also imposing length restrictions on precursor RNA, which are typically suitable only for sequences shorter than 500 nucleotides.^538,539^ Currently, researchers have proposed several solutions to these challenges. For example, optimizing synthesis methods and employing novel enzymatic strategies can improve the efficiency and quality of circRNA synthesis.^540–542^ Lee et al.^543^ have designed RNA constructs derived from Tetrahymena Group I introns that enable end to end self-targeting and splicing reactions to facilitate efficient circRNA generation. They have also optimized target sequence selection to enhance circularization efficiency. Additionally, enhancing the protein expression efficiency of circRNA remains a significant challenge in circRNA-related nanomedicine. Existing methods include RNA modifications, optimization of homologous arms, incorporation of internal ribosome entry sites (IRES), and the use of aptamers.^544–547^ Recently, a novel circRNA platform has been developed, incorporating an optimized vector topology, 5' and 3' untranslated regions (UTRs), IRES, and synthetic aptamers. This advancement has achieved a highly efficient and stable protein production process, with yields increased by several hundred times.^544,547^

Regarding screening techniques, existing methods must be continuously optimized to improve screening efficiency and accuracy while reducing costs. For instance, the development of more intelligent screening platforms capable of automatically adjusting screening parameters according to the characteristics of target molecules is necessary. While chemical modification strategies have improved the stability of nucleic acid aptamers, further exploration of more effective approaches remains imperative to address the requirements of diverse application scenarios. In terms of application fields, it is essential to further broaden the scope of applications for nucleic acid aptamers. Furthermore, in-depth investigations into the pharmacokinetics and biosafety of nucleic acid aptamers in vivo are required to establish a stronger foundation for their clinical applications. If these challenges can be addressed, exploring more valuable nucleic acid aptamers could signify a significant qualitative leap forward in this field’s research, potentially offering novel directions for future disease treatment strategies.

## Supplementary information

Supplementary information
