# Supplementary material for: Nucleic acid aptamers in orthopedic diseases: promising therapeutic agents for bone disorders
Source: Bone Res. 2025 Jul 24;13:71. doi: 10.1038/s41413-025-00447-8 (PMC12290010; doi:10.1038/s41413-025-00447-8)
Supplement: Supplementary file 1 — Supplementary information [file 41413_2025_447_MOESM1_ESM.docx]

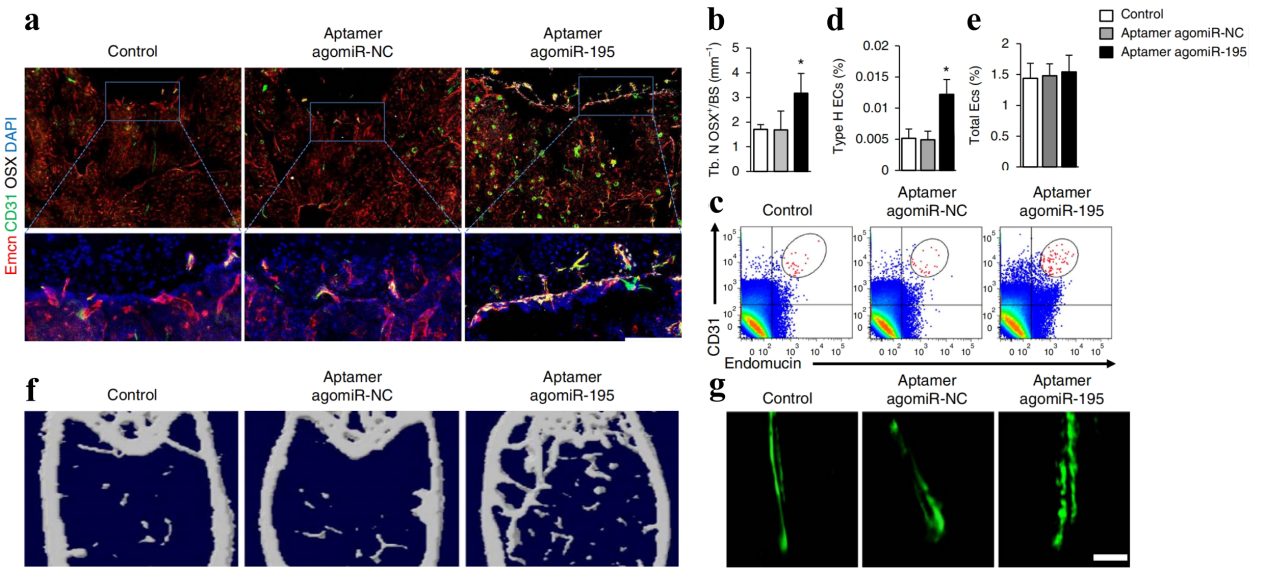


**Fig.** **S1** Injection aptamer-agomiR-195 enhanced CD31^hi^Emcn^hi^ vessel and bone formation. NC: negative control. **a** Representative images of immunostaining for CD31 (green), Emcn (red), and Osterix (white) in the murine femora treated with aptamer- agomiR-195 and their corresponding control group are presented. Scale bar, 100 μm. **b** Quantification of number of Osterix^+^ osteoprogenitors in distal femora. (n=5 murines in each group from three independent experiments). **c–e** FACS analysis dot plot **c** and quantitation of CD31^hi^Emcn^hi^ endothelial cells (Type H ECs) **d** and total endothelial cells (Total ECs) **e** from long bone of the aptamer-agomiR-195 treated murines. (n=6 murines in each group from three independent experiments). **f** Representative microcomputed tomography (μCT) images. **g** Representative images of calcein double labelling of trabecular bone. Data shown as mean±SD. *P＜0.05, (analysis of variance). Reproduced form ref.^299^ with permission from Nature Portfolio, copyright 2017.

**
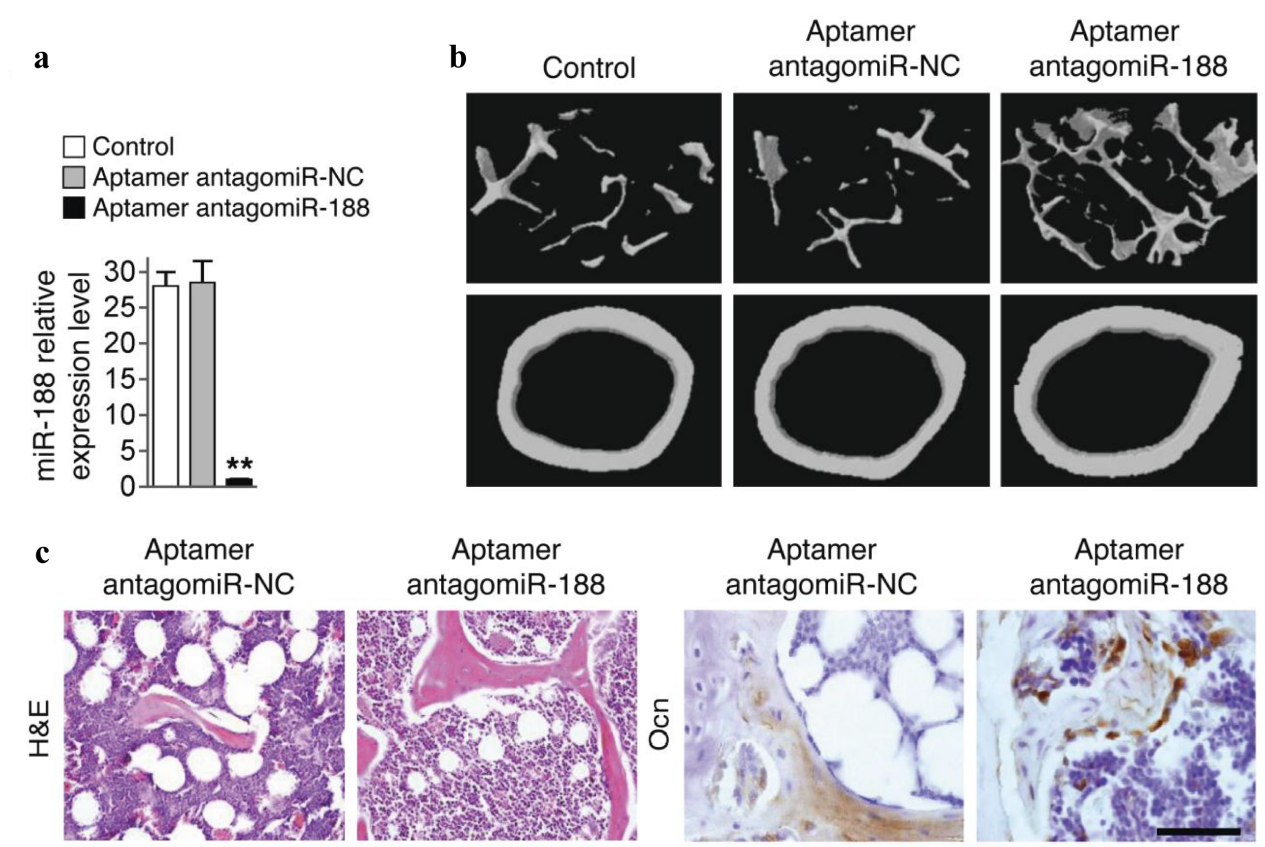
Fig. S2** Injection of aptamer-antagomiR-188 into bone marrow stimulates bone formation and decreases marrow fat accumulation in aged murines. **a** qRT-PCR analysis of the levels of miR-188 expression in murine BMSCs with BMSC-specific antagomiR-188 delivery. Aptamer-antagomiR-188 was injected into femoral bone marrow cavity of 15-month-old murines twice per month for 3 months. NC, negative control. **b** Representative μCT images. **c** Representative images of H&E staining and osteocalcin immunohistochemical staining. Reproduced form ref.^300^ with permission from the American Society for Clinical Investigation, copyright 2015.

**
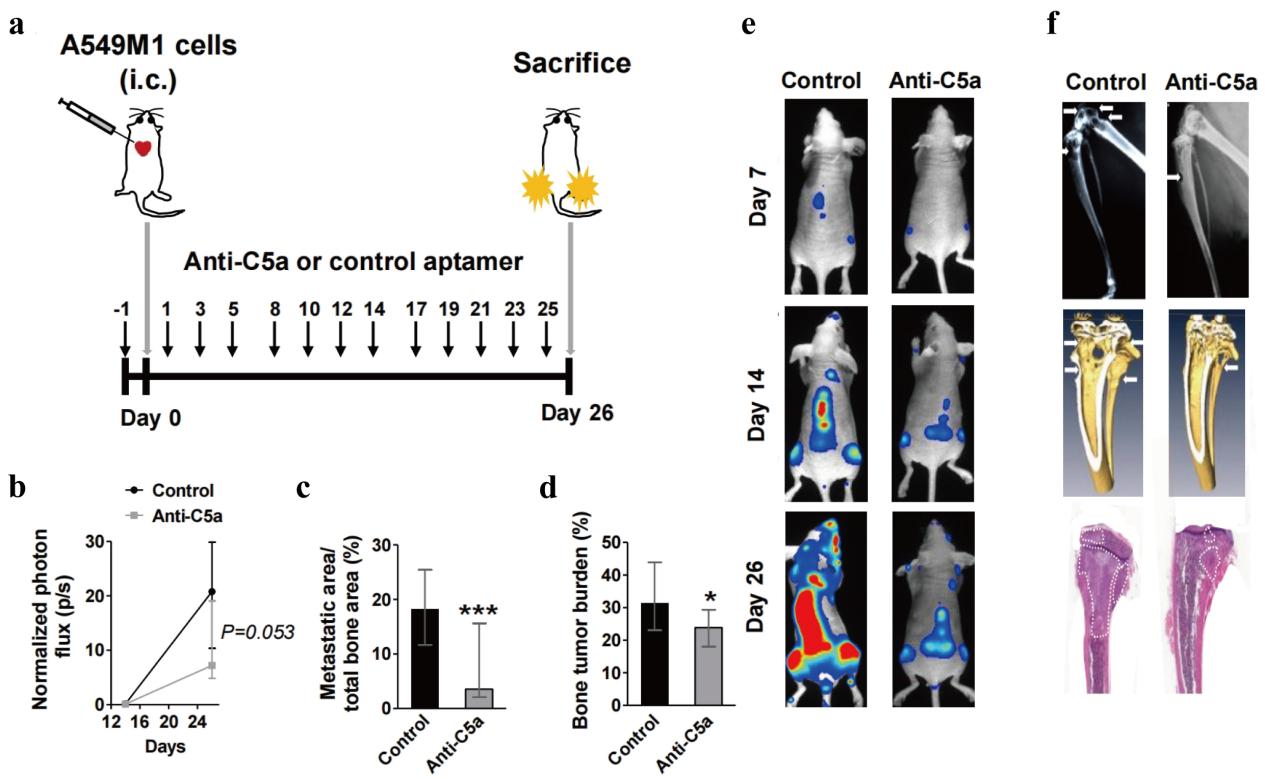
****Fig. S3** Effect of blocking C5a on bone metastasis formation of A549M1 cells. **a** Experimental regimen for the treatment of A549M1-injected murines with 10 mg/kg, i.p of the anti-C5a L-aptamer AON-D21 (n=7) or the control aptamer revAON-D21 (n=8). **b** Quantification of BLI in hindlimbs 26 days after i.c. inoculation. **c** Quantification of metastatic area by X-ray imaging. **d** Tumor burden assessed in H&E-stained sections. **e** Representative images of BLI (day 26). **f** Representative X-ray images (top row), µCT scans (middle row), and H&E-stained histologic sections (bottom row) of each group (day 26). Reproduced form ref.^337^ with permission from American Thoracic Society, copyright 2018.


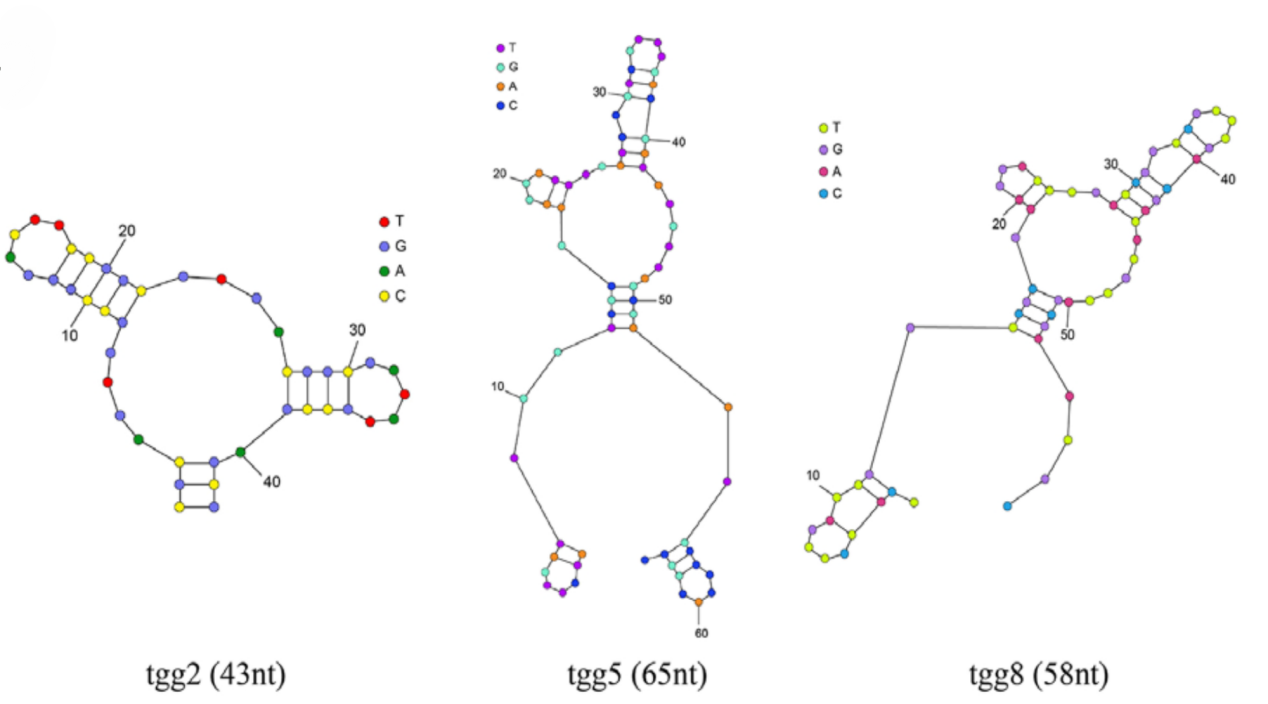


**Fig. S4** Proposed secondary structure of tgg2, tgg5 and tgg8. Reproduced form ref.^412^ with permission from [Elsevier](https://linkinghub.elsevier.com/retrieve/pii/S0003-4967(24)00529-6), copyright 2021.


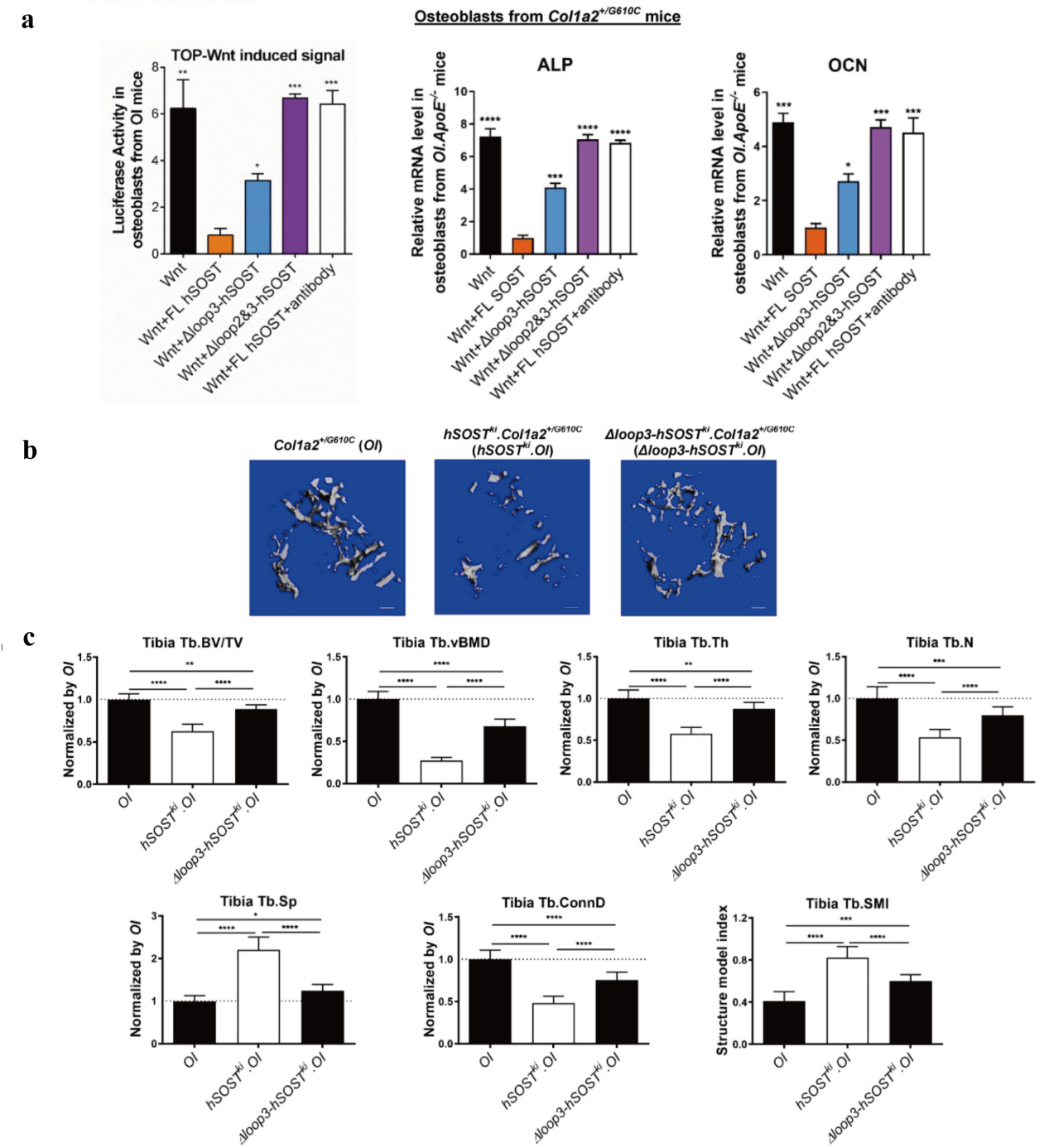


**Fig. S5** **a** Regulatory effects of FL hSOST, Δloop3-hSOST, Δloop2&3-hSOST, and FL hSOST combined with neutralizing antibody on Wnt signaling modulation and osteogenic differentiation capacity in Col1a2^+/G610C^-derived osteoblasts. **b** Representative 3D microarchitecture of proximal tibial metaphysis trabecular bone from three experimental groups: *Col1a2^+/G610C^* (*OI*), *hSOST^ki^.Col1a2^+/G610C^* (*hSOST^ki^*.*OI*), and *Δloop3-hSOST^ki^.Col1a2^+/G610C^* (*Δloop3-hSOST^ki^*.OI). Scale bars = 200 μm. **c** Quantitative micro-CT analysis of trabecular bone parameters including bone volume fraction (Tb.BV/TV), volumetric bone mineral density (Tb.vBMD), thickness (Tb.Th), number (Tb.N), trabecular spacing (Tb.Sp), connectivity density (Tb.conn.D) and structure model index (Tb.SMI) in the three experimental groups. Reproduced form ref.^512^ with permission from Ivyspring International Publisher, copyright 2022.


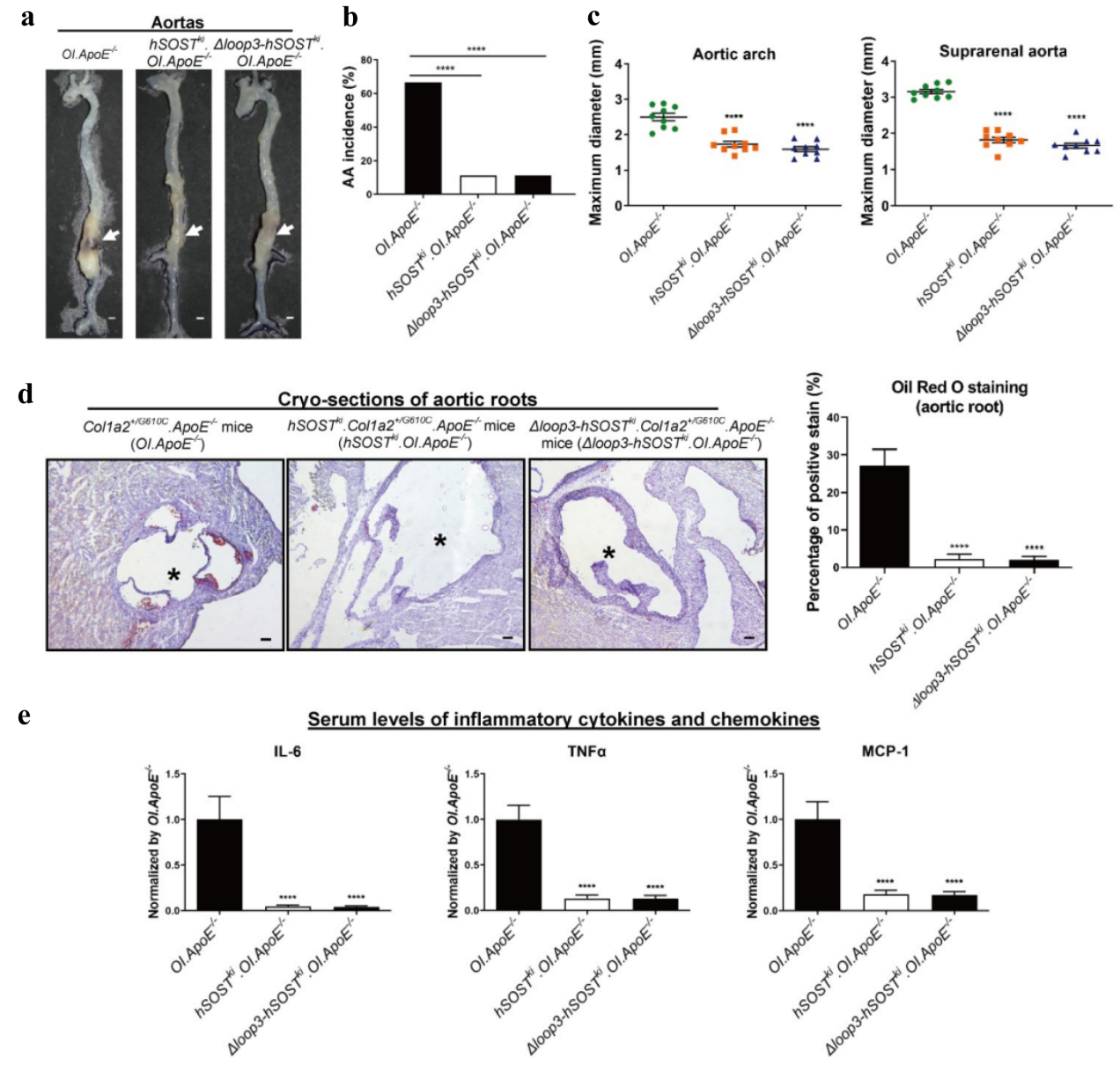


**Fig. S6** **a** Macroscopic evaluation of aortic morphology in experimental groups: *Col1a2^+/G610C^*.*ApoE^-/-^* (*OI.ApoE^-/-^*), *hSOST^ki^.Col1a2^+/G610C^*.*ApoE^-/-^* (*hSOST^ki^.OI.ApoE^-/-^*), and *Δloop3-hSOST^ki^.Col1a2^+/G610C^*.*ApoE^-/-^* (*Δloop3-hSOST^ki^*.*OI.ApoE^-/-^*). White arrows denote aneurysmal dilatations in the ascending aorta. Scale bars = 1 mm. **b** Comparative analysis of aortic aneurysm incidence among groups. Statistical significance determined by two-tailed Chi-square test. **** P<0.0001. **c** Morphometric quantification of maximum aortic dimensions: Left panel - thoracic aortic arch diameter; Right panel - suprarenal aorta diameter. **d** Atherosclerotic plaque characterization: Left - Representative Oil Red O-stained cryosections of aortic root. Scale bar = 100μm (*lumen). Quantitative assessment of plaque burden expressed as percentage of total vessel area. **e** Systemic inflammatory profiling showing serum concentrations of key mediators. Reproduced form ref.^512^ with permission from Ivyspring International Publisher, copyright 2022.
